# Complete genome sequence of *Corynebacterium variabile *DSM 44702 isolated from the surface of smear-ripened cheeses and insights into cheese ripening and flavor generation

**DOI:** 10.1186/1471-2164-12-545

**Published:** 2011-11-03

**Authors:** Jasmin Schröder, Irena Maus, Eva Trost, Andreas Tauch

**Affiliations:** 1 Institut für Genomforschung und Systembiologie, Centrum für Biotechnologie, Universität Bielefeld, Universitätsstraße 27, D-33615 Bielefeld, Germany; 2 CLIB Graduate Cluster Industrial Biotechnology, Centrum für Biotechnologie, Universität Bielefeld, Universitätsstraße 27, D-33615 Bielefeld, Germany

## Abstract

**Background:**

*Corynebacterium variabile *is part of the complex microflora on the surface of smear-ripened cheeses and contributes to the development of flavor and textural properties during cheese ripening. Still little is known about the metabolic processes and microbial interactions during the production of smear-ripened cheeses. Therefore, the gene repertoire contributing to the lifestyle of the cheese isolate *C. variabile *DSM 44702 was deduced from the complete genome sequence to get a better understanding of this industrial process.

**Results:**

The chromosome of *C. variabile *DSM 44702 is composed of 3, 433, 007 bp and contains 3, 071 protein-coding regions. A comparative analysis of this gene repertoire with that of other corynebacteria detected 1, 534 predicted genes to be specific for the cheese isolate. These genes might contribute to distinct metabolic capabilities of *C. variabile*, as several of them are associated with metabolic functions in cheese habitats by playing roles in the utilization of alternative carbon and sulphur sources, in amino acid metabolism, and fatty acid degradation. Relevant *C. variabile *genes confer the capability to catabolize gluconate, lactate, propionate, taurine, and gamma-aminobutyric acid and to utilize external caseins. In addition, *C. variabile *is equipped with several siderophore biosynthesis gene clusters for iron acquisition and an exceptional repertoire of AraC-regulated iron uptake systems. Moreover, *C. variabile *can produce acetoin, butanediol, and methanethiol, which are important flavor compounds in smear-ripened cheeses.

**Conclusions:**

The genome sequence of *C. variabile *provides detailed insights into the distinct metabolic features of this bacterium, implying a strong adaption to the iron-depleted cheese surface habitat. By combining *in silico *data obtained from the genome annotation with previous experimental knowledge, occasional observations on genes that are involved in the complex metabolic capacity of *C. variabile *were integrated into a global view on the lifestyle of this species.

## Background

Cheese is one of the oldest dairy products and generally based on the addition of rennet and lactic acid bacteria to milk of cows, sheep, goats, or buffalos. Nowadays, cheese is also the most diverse group of dairy products with hundreds of varieties that are capable of being differentiated mostly by their type of ripening [1]. Bacterial smear-ripened cheeses, such as Appenzeller, Gubbeen, Limburger, Livarot, Munster, and Tilsit, are characterized by the development of a viscous, red-orange smear on the surface of the cheese body during ripening. This smear is a microbial mat composed of various species of yeasts and bacteria. Their combined metabolic activities are mainly responsible for the typical flavor and textural properties of this type of cheese [2,3]. The microbiology of the red-orange smear is poorly understood so far. Generally, acid-tolerant yeasts grow during the first days of ripening of smear-ripened cheeses and metabolize the lactic acid produced by the lactic starter cultures to CO_2 _and H_2_O, thereby increasing the pH and producing growth factors, such as pantothenic acid, that permit the growth of Gram-positive and Gram-negative bacteria [4,5]. Yeast species can also deaminate amino acids to ketoacids and NH_3_, which leads to deacidification of the cheese curd in such a way that bacteria are able to grow [6,7]. The bacterial microflora dominating the later stages of ripening of smear-ripened cheeses is composed of salt-tolerant micrococci, staphylococci, and corynebacteria [2,8]. In the past, it was supposed that the coryneform species *Brevibacterium linens *represents the major organism on the surface of smear-ripened cheeses [9], but recent investigations emphasized the importance of other coryneform bacteria to the ripening process and identified *Arthrobacter nicotianae*, *Arthrobacter arilaitensis*, *Brevibacterium ammoniagenes*, *Microbacterium gubbeenense*, *Rhodococcus fascians*, and members of the genus *Corynebacterium *(*Corynebacterium ammoniagenes*, *Corynebacterium casei*, and *Corynebacterium variabile*) dominating the microflora of Gubbeen, Tilsit, and other smear-ripened cheeses [10-14].

The genus *Corynebacterium *includes (besides numerous human and animal pathogens) a diverse collection of non-pathogenic species that have been detected in a wide variety of habitats, such as soil, plant material, waste water, and dairy products [15]. *C*. *casei*, for instance, belongs to the main lineage of the genus *Corynebacterium *[4,16] and was identified as the dominant species on the surface of a Gubbeen-type Irish farmhouse smear-ripened cheese by taxonomic analyses [12,17]. The abundance of *C*. *casei *on the cheese surface is closely followed by *C. variabile*, which is taxonomically located in a distinct subline (cluster 3) of the genus *Corynebacterium*, with *Corynebacterium jeikeium *and *Corynebacterium urealyticum *as the most prominent phylogenetic relatives [18,19]. *C. casei *and *C. variabile *are salt-tolerant and able to grow in the presence of 8.0% NaCl and at pH values below 4.9 [17]. Both corynebacteria also metabolize the lactate produced by the starter bacteria, implying that they can grow from the beginning of cheese ripening and may therefore not depend on significant yeast growth during the initial stages of the ripening process [17].

In the present study, we characterize the complete genome sequence of *C. variabile *DSM 44702 (formerly *Corynebacterium mooreparkense*) that was originally isolated from the smear-ripened cheese Gubbeen and identified by 16S rDNA sequencing and DNA-DNA hybridization studies [4]. It was initially described as the type strain of a separate species named *C. mooreparkense *because the organism was isolated at the Dairy Products Research Centre in Moorepark, Ireland [4]. However, additional molecular taxonomic studies demonstrated considerable similarities of all *C. mooreparkense *isolates with the species *C. variabile *at the levels of 16S rDNA gene sequence and DNA-DNA relatedness [20]. It was therefore concluded that the name *C. mooreparkense *is a later heterotypic synonym of *C. variabile *[20]. In the present study, the annotation of the complete genome sequence and the deduced genetic repertoire of *C. variabile *DSM 44702 provide detailed insights into the lifestyle and metabolic features of this species, which is strongly adapted to the cheese surface habitat, where it contributes to flavor and texture of the final product.

## Results

### Pyrosequencing and annotation of the *C. variabile *DSM 44702 genome

**Figure 1:**
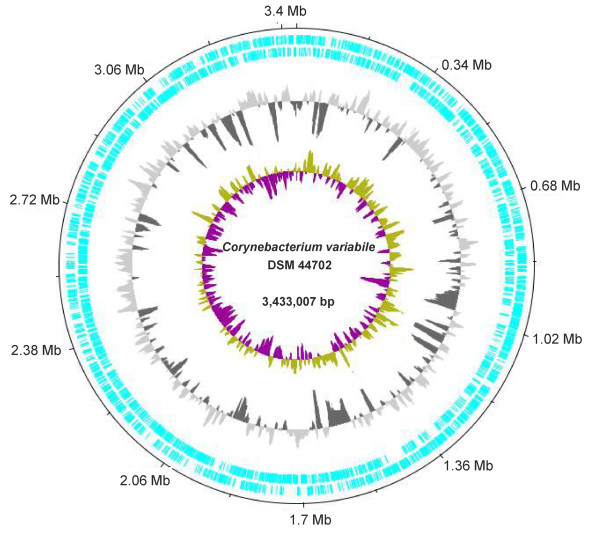
***Corynebacterium variabile *DSM 44702 genome plot**. The circles represent from the outside: circle 1, DNA base position [Mb]; circle 2, protein-coding regions transcribed clockwise; circle 3, protein-coding regions transcribed anticlockwise; circle 4, G+C content plotted using a 10-kb window; circle 5, G/C skew plotted using a 10-kb window. The genome plot of *C. variabile *DSM 44702 was generated with the web version of the DNAPlotter tool.

**Figure 2:**
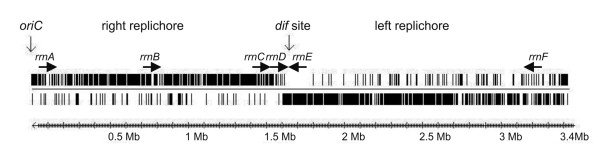
**Distribution of architecture imparting sequences in the *C. variabile *DSM 44702 chromosome**. The distribution of the octamer sequences G(A/T/C)GGGGGA and (T/C)GGGGGAG on the leading and lagging strands of the *C. variabile *chromosome is shown. The origin of chromosomal replication (*oriC*) is marked. The deduced *dif *site is located at 1.603 Mb of the chromosomal map. The location of six rRNA operons (*rrnA*-*rrnF*) on the leading strands of the *C. variabile *chromosome is indicated.

The DNA sequence of the *C. variabile *DSM 44702 chromosome was determined by a whole-genome shotgun approach using pyrosequencing. A quarter of a sequencing run with the Genome Sequencer FLX Instrument and Titanium chemistry yielded 253, 845 reads and 97, 765, 747 bases that were assembled into 93 large contigs (≥500 bases) and 41 small contigs, indicating numerous repetitive elements and insertion sequences in the *C. variabile *DSM 44702 genome. The remaining gaps in the chromosomal sequence were closed by PCR strategies, supported by the r2cat tool [21] and the Consed program [22]. The final assembly of the DNA sequences yielded a circular chromosome with a size of 3, 433, 007 bp (Figure 1). A 28-fold coverage was thus obtained by pyrosequencing when considering the final size of the *C. variabile *DSM 44702 chromosome. The mean G+C content of the *C. variabile *DSM 44702 genome is 67.15%, which is significantly higher than the value (60%) initially determined by a reverse-phase HPLC method [4]. The annotation of the *C. variabile *genome sequence was performed with the GenDB software system [23] and resulted in the detection of 3, 071 protein-coding regions. Furthermore, 59 tRNA genes were predicted by the tRNAscan-SE program [24] and six *rrn *operons were detected on the leading strands of the *C. variabile *chromosome (Figure 2).

### General architecture of the *C. variabile *DSM 44702 genome

The plot of the calculated G/C skew [(G-C)/(G+C)] indicated a typical bi-directional replication mechanism of the *C. variabile *chromosome (Figure 1). According to the presence and distribution of six conserved DnaA boxes, the origin of chromosomal replication (*oriC*) is located downstream of the *dnaA *coding region [25]. Moreover, the biased distribution of architecture imparting sequences (AIMS) on the leading strands of the *C. variabile *chromosome indicated the presence of a *dif *region [26] at position 1, 603 kb on the chromosomal map, dividing the chromosome of *C. variabile *into two replichores of nearly similar size (Figure 2). Due to the highly biased presence of AIMS on the leading strands of the *C. variabile *chromosome (Figure 2), we also examined the gene-strand bias in this strain. In total, 59.6% of the predicted gene repertoire is located on the leading strands of the *C. variabile *chromosome, revealing a moderate gene-strand bias in this species. However, it has been suggested previously that essentiality is the driving force for gene-strand bias in bacterial genomes [27]. We therefore calculated also the gene-strand bias for candidate essential genes in *C. variabile*. For this purpose, the set of 658 candidate essential genes detected in the genome of *Corynebacterium glutamicum *R by high-density transposon mutagenesis [28,29] was compared to the predicted gene repertoire of *C. variabile *by reciprocal BLASTP matches with the EDGAR software [30]. This comparative content analysis revealed 373 of the candidate essential genes from *C. glutamicum *R having orthologs in the *C. variabile *genome. The majority of these genes (80.4%) are located on the leading strands of the *C. variabile *chromosome, with 68% of all candidate essential genes being located on the left replichore.

**Figure 3:**
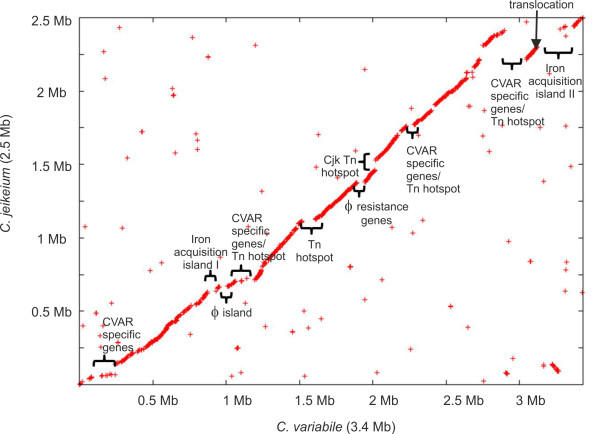
**Synteny between the chromosomes of *C. variabile *DSM 44702 and *C. jeikeium *K411**. The X-Y plot is composed of dots forming syntenic regions between both chromosomes. Each dot represents a predicted *C. variabile *protein having an ortholog in the *C. jeikeium *chromosome, with co-ordinates corresponding to the position of the respective coding region in each genome. Orthologous proteins were identified by reciprocal best BLASTP matches with the EDGAR software. The genomic region of *C. variabile *DSM 44702 indicating a translocation event is marked by an arrow. Selected genomic regions characterized by breakpoints of synteny between *C. variabile *and *C. jeikeium *are labeled according to their predicted gene content.

Synteny analysis by reciprocal best BLASTP hits [30] revealed a highly conserved order of orthologous genes between the chromosomes of *C. variabile *DSM 44702 and *C. jeikeium *K411, with the exception of a translocated DNA region comprising a segment of approximately 125 kb in size (Figure 3). This result is consistent with the very close phylogenetic relationship of both species that belong to the same subline (cluster 3) of the genus *Corynebacterium *[18,19], and the general observation that genetic rearrangements are rare in corynebacterial genomes [15,31]. The genome of *C. variabile *DSM 44702 thus contains the previously predicted inversions around *dif *and *oriC*, which are both characteristic genomic features of cluster 3 species in the genus *Corynebacterium *[31]. However, the synteny between both genomes is interrupted due to the presence of additional genes in the *C. variabile *chromosome that were functionally assigned to a phage island, several transposon hotspots, and species-specific gene islands, the latter containing for instance large gene clusters involved in iron acquisition and siderophore biosynthesis (Figure 3). Some of these species-specific gene islands show a significant deviation from the mean G+C content of the *C. variabile *chromosome and are flanked by several insertion sequences, suggesting that horizontal gene transfer contributed to the evolution of the current gene repertoire of strain DSM 44702 (Figure 1).

The phage island of the *C. variabile *chromosome has a size of about 48.3 kb and comprises 60 genes (CVAR_0826-CVAR_0886). This genomic region is locted between two tRNA^Gly ^genes that may represent the integration site of a putative phage. This view is supported by the presence of 50-bp direct repeats, which are part of the tRNA genes and flank the prophage-like region. Most genes (39 out of 60) of the phage island encode hypothetical proteins of unknown function, whereas others encode enzymes involved in phage DNA replication, recombination, and repair. The structural proteins of the putative phage revealed similarities to the tail structure of *Rhodococcus *phage ReqiPine5 [32] and to the capsid structure of *Listeria *phage A006 [33].

**Figure 4:**
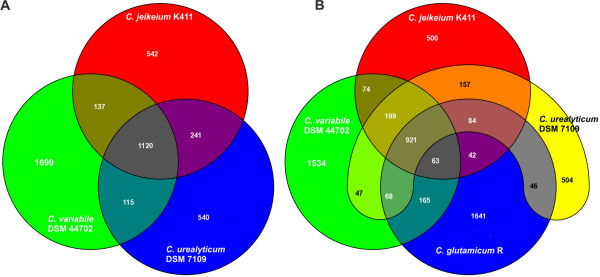
**Comparative content analysis of the gene repertoire of *C. variabile *DSM 44702 and other corynebacteria**. **(A)**, Venn diagram showing the comparison between the genomes of *C. variabile *DSM 44702, *C. jeikeium *K411, and *C. urealyticum *DSM 7109, all belonging to the cluster 3 subline of the genus *Corynebacterium*. **(B)**, Venn diagram showing the comparison of *C. variabile *DSM 44702, *C. jeikeium *K411, *C. urealyticum *DSM 7109, and *C. glutamicum *R, the latter being a member of the main lineage of the genus *Corynebacterium*. This comparative content analysis exploits all protein-coding regions of the selected genomes and detects orthologs by reciprocal best BLASTP matches with the EDGAR software.

The calculated reciprocal best BLASTP hits [30] were moreover used to compare the predicted proteome of *C. variabile *DSM 44702 with the complete set of proteins encoded in the genomes of *C. jeikeium *K411 [34] and *C. urealyticum *DSM 7109 [31], which are close taxonomic relatives with known genome sequences. This comparative content analysis at proteome level revealed that 1, 120 proteins (36.5%) of *C. variabile *share homologs in the genomes of *C. jeikeium *and *C. urealyticum *(Figure 4A). According to this comparative data, *C. variabile *contains 1, 699 proteins with no homologous counterparts in the proteomes of the taxonomic relatives *C. jeikeium *and *C. urealyticum*. However, it has to be considered that the three genomes of the taxonomically closely related species differ considerably in their sizes as well as in the numbers of protein-coding regions [31,34]. Thus, the 3.31 Mb sequence data of the soil bacterium *C. glutamicum *R [28] was incorporated into this comparative analysis, revealing a comparably extensive set of 1, 534 genes that are specific for *C. variabile *when compared to the selected corynebacteria (Figure 4B). This calculation indicates the presence of an extensive and still unexplored variability in the gene equipment of non-pathogenic corynebacteria. This set of genes might contribute to the characteristic features of *C. variabile *that define its distinct metabolic capabilities.

In the following sections, we present the detailed analysis of these metabolic features of *C. variabile *DSM 44702 and combine them with data deduced from the predicted transcriptional regulatory repertoire. This approach in evaluating the complete genome sequence of *C. variabile *DSM 44702 revealed a collection of relevant genes contributing to the lifestyle of this species and their integration into a transcriptional gene-regulatory network.

### General metabolic features of *C. variabile *DSM 44702 deduced from the complete genome sequence

The experimental work on the taxonomic description of *C. variabile *DSM 44702 as the type strain of the former species *C. mooreparkense *revealed the ability of this cheese isolate to utilize glucose, fructose, mannose, and ribose as carbon and energy sources [4]. Bioinformatic analysis of the gene repertoire assigned to the central carbohydrate metabolism detected a complete set of genes involved in glycolysis, gluconeogenesis, and the pentose phosphate pathway, as well as the presence of glucose- and fructose-specific components (*ptsG *and *ptsF*) of the bacterial phosphoenolpyruvate:carbohydrate phosphotransferase system (*ptsH *and *ptsI*) and at least one ABC-type transport system (*sugABCD*) for sugar uptake (Additional file 1). The *stcRS *genes of a two-component signal transduction system are located directly upstream of the putative *sugABCD *operon and might be involved in the transcriptional control of this sugar importer. Glucokinase and ribokinase genes (*glk *and *rbsK*) are present in *C. variabile *DSM 44702, allowing the conversion of "free" sugars into phosphorylated central pathway intermediates (Additional file 1).

According to the genome annotation, *C. variabile *DSM 44702 can utilize gluconate that is imported by gluconate permease (*gntP*) and converted to 6-phosphogluconate by gluconate kinase (*gntK*) (Additional file 1). *C. variabile *DSM 44702 can moreover channel propionate via its methylcitrate cycle genes [35] into the tricarboxylic acid cycle (Additional file 1). Calcium propionate occurs naturally in many dairy products including cheese, and some types of cheese contain as much as 1% of natural propionic acid [36]. Propionate is imported into *C. variabile *DSM 44702 by a monocarboxylic acid transporter (*mctC*) [37]. This secondary transport system can probably also import pyruvate that exists naturally in cheese in small amounts [37,38].

The tricarboxylic acid cycle of *C. variabile *DSM 44702 and the glyoxylate bypass are complete, with the exception of the *sucCD *genes encoding two subunits of succinyl-CoA synthetase. The absence of these genes was also observed in other *Corynebacterium *species, such as *C. diphtheriae *[39], *C. jeikeium *[34], and *C. urealyticum *[31]. It has been suggested that a succinyl-CoA:CoA transferase (*cat1*) catalyzes the conversion of succinyl-CoA to succinate instead [39,40]. Candidate succinyl-CoA:CoA transferases are encoded by the neighboring paralogs *cat1 *(CVAR_0586) and *cat2 *(CVAR_0587) in the *C. variabile *DSM 44702 chromosome. Anaplerotic reactions in *C. variabile *DSM 44702 are accomplished by pyruvate carboxylase (*pyc*) and phosphoenolpyruvate carboxylase (*ppc*).

All components constituting a non-branched respiratory chain are present in *C. variabile *DSM 44702, along with complete menaquinone and heme biosynthesis pathways (Additional file 2). The terminal oxidase of the respiratory chain most likely consists of both cytochrome *bc*1 oxidase (*qcrCAB*) and cytochrome *aa*3 oxidase (*ctaCDEF*) that might constitute a cytochrome *bc*1-*aa*3 supercomplex, as previuosly described for *C. glutamicum *[41]. Moreover, the genome of *C. variable *DSM 44702 contains the *cydAB *genes encoding the subunits of cytochrome *bd *oxidase. In mycobacteria, the cytochrome *bd *oxidase is induced under microaerobic conditions because of the high oxygen affinity of this enzyme [42]. This suggests that the cytochrome *bc*1-*aa*3 supercomplex is of basic importance for *C. variabile *DSM 44702 under aerobic growth conditions. The genes encoding subunits of the F_1_F_0_-ATP synthase complex that is essential for ATP generation by oxidative phosphorylation are present and organized in a putative operon (*atpBEFHAGDC*).

The genome sequence of *C. variabile *DSM 44702 was moreover screened for genes encoding enzymes, which degrade, modify, or create glycosidic bonds according to data stored in the CAZy database [43]. This seed information of CAZy includes glycoside hydrolases, glycosyl transferases, and carbohydrate esterases. The content of the CAZy database also covers the associated carbohydrate-binding module, comprising carbohydrate-active enzymes that often display a modular structure with the aforementioned proteins. Candidate carbohydrate-active enzymes encoded in *C. variabile *DSM 44702 were identified by a combined approach using BLASTp analysis of all corynebacterial enzymes stored in the CAZy database and hidden Markov models [44] created from amino acid sequence alignments downloaded from the Pfam database [45]. Glycoside hydrolases (GH) hydrolyze glycosidic bonds and are a widespread group of enzymes with at least 128 families [43,46]. *C. variabile *DSM 44702 encodes 15 candidate glycoside hydrolases associated to eight families. The protein families GH13, GH23, and GH25 are represented by three proteins each, whereas the families GH32, GH65, GH76, and GH19 contain only a single protein member. Two proteins of *C. variabile *DSM 44702 were associated to the glycoside hydrolase family GH3. Glycosyl transferases (GT) catalyze the transfer of sugar moieties during polysaccharide biosynthesis and are classified into 94 families [43,47]. A total number of 28 candidate glycosyl transferases was detected in the genome of *C. variabile *DSM 44702 and grouped into 13 protein families. The largest families are GT2 and GT4, each containing six proteins, followed by family GT87 with three enzymes, and GT1, GT3, and GT51 with two members. One protein was identified in each of the families GT5, GT20, GT28, GT35, GT39, GT53, and GT85. Carbohydrate esterases (CE) catalyze the de-*O*-acylation or de-*N*-acylation of substituted saccharides and are grouped into 16 families [43,48]. *C. variabile *DSM 44702 encodes 14 candidate carbohydrate esterases, six of them grouped into family CE1 and six enzymes classified into family CE9. Family CE14 contains two proteins. A carbohydrate-binding module (CBM) is defined as a contiguous amino acid sequence within a carbohydrate-active enzyme with a discreet fold having carbohydrate-binding activity [49]. In *C. variabile *DSM 44702, two proteins were assigned to family CBM48, but the respective proteins are simultaneously associated to family GH13. Altogether, 57 candidate proteins constitute the set of carbohydrate-active enzymes of *C. variabile *DSM 44702.

Moreover, the hitherto known biosynthesis pathways for vitamins and cofactors in corynebacteria were reconstructed and shown to be complete in *C. variabile *DSM 44702, including the synthesis of molybdenum cofactor (Additional file 2). The anabolic pathways for purine and pyrimidine nucleotides are also complete (data not shown), as are all pathways for the *de novo *biosynthesis of standard proteinogenic amino acids (Additional file 3). The *C. variabile *DSM 44702 genome is equipped with several transport systems allowing the import of "free" amino acids (Additional file 3). The content of amino acids varies between different types of cheese mainly due to the duration and intensity of proteolysis during the ripening process [50]. Seven amino acids are often concentrated in cheese and contribute to the taste of the final product: leucine, lysine, and phenylalanine (bitter); proline and valine (bitter sweet); aspartate and glutamate (salty umami) [50]. According to the detailed knowledge of amino acid transporters in *C. glutamicum*, putative importers for these amino acids are encoded in *C. variabile *DSM 44702 by *brnQ *(leucine, valine, and isoleucine [51]), *lysI *(lysine [52]), *pheP *(phenylalanine [53]), *aroP *(phenylalanine, tyrosine, and tryptophan [54]), *proP *and *putP *(proline [55,56]), and *gluABCD *(glutamate [57]) (Additional file 3). In addition, the sulphur-containing amino acid methionine, which is the precursor of the flavor compound methanethiol, is imported into *C. variabile *DSM 44702 by two transport systems, an ABC-type transporter encoded by the *metQNI *genes and a two-subunit member of the neurotransmitter:sodium symporter family encoded by the *metP*-*metPS *gene pair [58] (Additional file 3).

### The secretome of *C. variabile *DSM 44702 deduced from the complete genome sequence

Like other members of the genus *Corynebacterium*, *C. variabile *DSM 44702 encodes two types of protein secretion systems, the general secretory (Sec) pathway and the twin-arginine translocation (Tat) pathway. The Sec translocation pathway has two distinct functions in bacterial protein trafficking, the cotranslational integration of proteins into the cytoplasmic membrane and the posttranslational translocation of proteins across the membrane [59]. The posttranslational export of proteins is facilitated by the SecYEG translocon in conjunction with the multifunctional protein SecA. The latter protein binds to cytosolic precursor proteins destined for export and delivers them to the SecYEG translocon. As the SecA protein is also an ATPase, it provides energy for the stepwise translocation of the precursor protein through the channel of the SecYEG translocon. The precursor proteins have a typical signal sequence at the amino-terminal end, which is cleaved from the precursor by either signal peptidase I or signal peptidase II [59].

The genome sequence of *C. variabile *DSM 44702 encodes all protein components of the basic Sec machinery necessary for protein secretion. The *lepB1 *and *lepB2 *genes encode homologs of signal petidase I (EC 3.4.21.89), whereas the *lspA *gene codes for signal petidase II, also named lipoprotein signal peptidase (EC 3.4.23.36). In addition, the *secA2 *gene of *C. variabile *DSM 44702 provides a homolog of the preprotein translocase subunit SecA. The presence of the accessory protein SecA2 was reported previously for other Gram-positive bacteria. In *M. tuberculosis *and *M. smegmatis*, the SecA2 protein is required for the export of a small subset of exoproteins, whereas SecA1 remains the essential housekeeping translocase [60]. On the other hand, both SecA homologs are probably essential for viability in *C. glutamicum *[61]. *C. variabile *DSM 44702 moreover encodes the signal recognition particle SRP, which is essential for targeting of almost all inner membrane proteins in *E. coli *[62], as well as its receptor FtsY [59].

To estimate the number of secreted proteins encoded by *C. variabile *DSM 44702, the first 70 amino acid residues of each protein were used to search for amino-terminal signal peptides with SignalP 4.0 [63]. In this way, a total number of 363 proteins were identified to be potentially secreted by *C. variabile*. To furtheron deduce the number of putative lipoproteins, all secreted proteins were screened for the presence of a lipobox motif using the DOLOP database tools [64]. Altogether, 66 lipobox-containing proteins were identified in the proteome of *C. variabile *DSM 44702. The signal peptides of these proteins are therefore most likely recognized by signal peptidase II. The signal peptides of the remaining 297 proteins are recognized by signal peptidase I and the respective proteins are exported by the machinery of the Sec pathway of *C. variabile *DSM 44702. However, it has to be considered that proteins with membrane-spanning domains might also contain signal peptides that are recognized by type I signal peptidases [65]. Out of the 363 proteins predicted to be secreted by *C. variabile *DSM 44702, a set of 100 proteins with membrane-spanning domains were detected by the TMHMM tool [66], which therefore may be destined for the integration into the cytoplasmic membrane. This sub-set of proteins comprises eleven membrane-associated enzymes with functions in the respiratory chain or in cell wall turnover of *C. variabile *DSM 44702 and 25 putative permeases with transport functions for diverse substrates. Two-thirds of the proteins were annotated as putative membrane proteins with unknown functions. After exclusion of proteins with membrane-spanning domains, the remaining 263 predicted extracellular proteins of *C. variabile *DSM 44702 were grouped into functional categories according to the classification scheme of the Clusters of Orthologous Groups of proteins (COG) [67]. A large portion of the predicted extracellular proteins was assigned to COG classes R (16%), S (6.4%), and X (39.8%), containing proteins that are currently not grouped into COGs or whose precise functions are hitherto unknown. Prominent functional classes of the extracellular proteins of *C. variabile *DSM 44702 comprise inorganic ion transport and metabolism (COG class P; 16%), cell wall and membrane biogenesis (COG class M; 7.6%), and signal transduction mechanisms (COG class T; 3.4%).

The Tat pathway of *C. variabile *DSM 44702 is represented by two members of the TatC protein family encoded by *tatC1 *and *tatC2*, as well as four members of the TatA family encoded by paralogous versions of *tatA *and *tatB *[68]. Proteins are targeted to the Tat pathway by N-terminal signal peptides containing the twin-arginine motif that is followed by a hydrophobic stretch of amino acids [69]. In *C. variabile *DSM 44702, a total number of ten proteins containing signal peptides meeting these criteria were detected by the prediction tools TATP [70] and TATFIND [71]. This small number of proteins secreted by the Tat pathway is comparable to those predicted previously in *C. diphtheriae *[72], *C. glutamicum*, and *C. efficiens *[65,73]. The set of proteins secreted by the Tat pathway of *C. variabile *DSM 44702 includes for instance an alkaline phosphatase (EC 3.1.3.1), a ceramidase (EC 3.5.1.23), an esterase of the SGNH type, and a Dyp-type peroxidase.

### The transcriptional regulatory repertoire of *C. variabile *DSM 44702 deduced from the complete genome sequence

**Figure 5:**
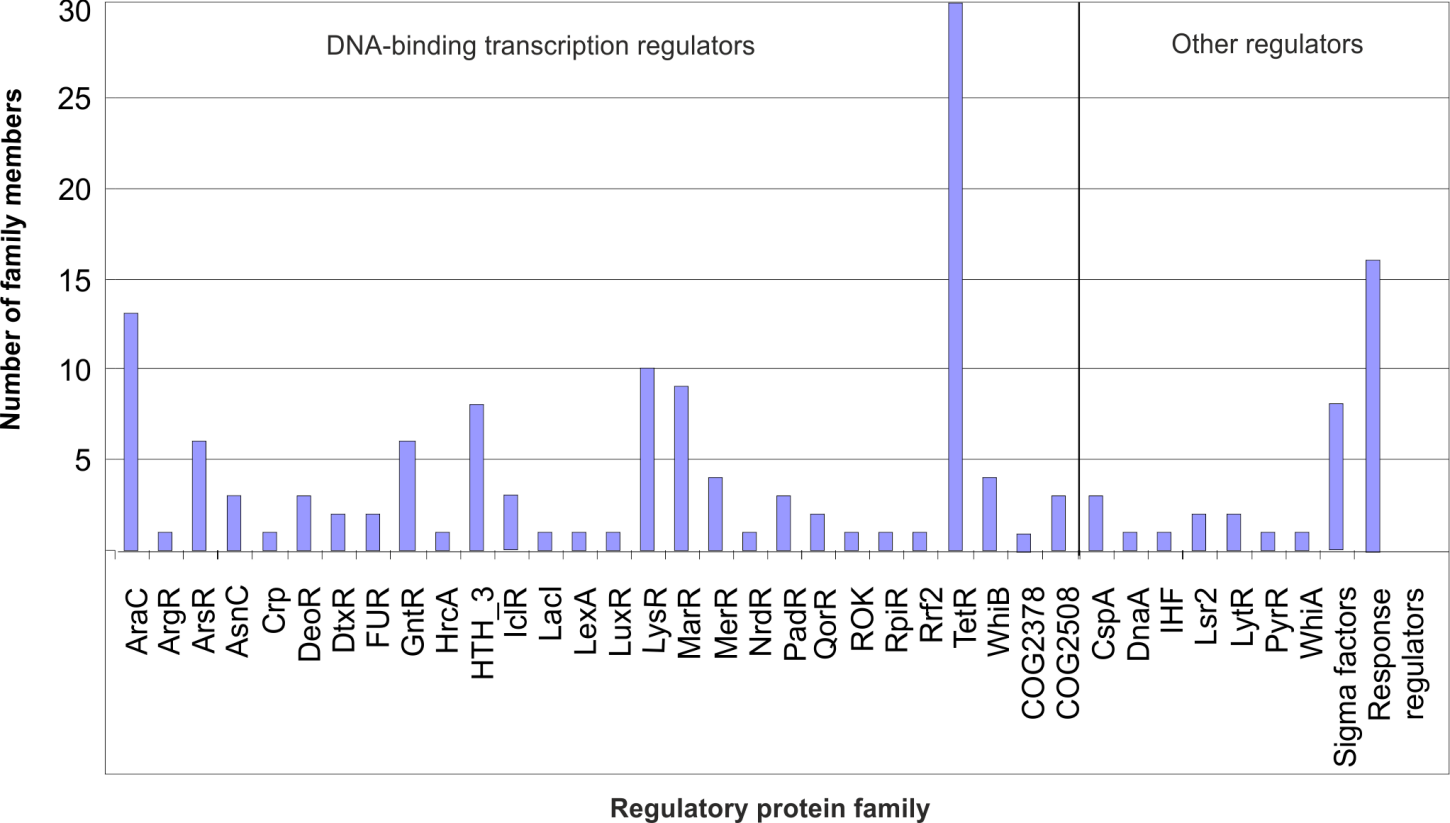
**Classification of the transcriptional regulatory repertoire of *C. variabile *DSM 44702 into regulatory protein families**. The number of candidate transcription regulators assigned to a regulatory protein family is shown.

The repertoire of candidate transcription regulators encoded in the *C. variabile *DSM 44702 genome was deduced from the functional genome annotation, taking into account the comprehensive knowledge of the gene-regulatory network of *C. glutamicum *ATCC 13032 [74]. A collection of 156 genes encoding DNA-binding transcription regulators (132 genes), sigma factors (8 genes), and response regulators of two-component signal transduction systems (16 genes) can be regarded as the minimal regulatory repertoire of *C. variabile *DSM 44702 (Figure 5). It is noteworthy that among the genes for response regulators, four are not associated with a corresponding sensor histidine kinase gene (CVAR_0325, CVAR_0577, CVAR_1009, and CVAR_2072). The deduced set of candidate transcription regulators represents 5.1% of the predicted protein-coding genes of the *C. variabile *genome. This value is in the range known from the 3.28 Mb genome of *C. glutamicum *ATCC 13032 (5.3%) [74] and is in agreement with previous observations that less than 10% of the total number of predicted proteins is associated with transcriptional regulatory processes in bacteria [75].

The collection of potential transcription regulators was grouped into regulatory protein families according to their amino acid sequence similarities and domain organizations [76]. This bioinformatic classification assigned the predicted transcription regulators of *C. variabile *to 37 regulatory protein families that vary significantly in their number of representatives (Figure 5). The largest family of DNA-binding transcription regulators is TetR with 30 members, followed by AraC with 13 proteins. The TetR family of transcription regulators is widely distributed among bacterial species [77] and is also the most prevalent group of regulatory proteins in other corynebacteria [78]. The AraC family of transcription regulators comprises a diverse group of proteins that are involved in the regulation of various biological processes, such as carbon and nitrogen metabolism, adaptive responses, stress responses, and virulence [79]. The number of AraC-type transcriptional regulators is remarkably high in *C. variabile*, as other non-pathogenic corynebacteria encode only three or four members of this protein family, whereas genes encoding AraC-type regulators are less prevalent or even absent in the genomes of pathogenic corynebacteria [78,80,81].

*C. variabile *encodes only 22 proteins of the previously detected collection of 24 transcription regulators that constitute the core set of DNA-binding transcription regulators in corynebacteria. The genome of *C. variabile *DSM 44702 lacks orthologs of the conserved regulators RamB and SugR that are involved in the transcriptional control of acetate metabolism and central sugar metabolism, respectively [74,78]. To detect regulatory interactions contributing to metabolic pathways relevant for cheese ripening and flavor generation by *C. variabile*, the deduced knowledge of the transcriptional regulatory repertoire was combined with bioinformatic motif searches for DNA-binding sites of prominent regulatory proteins [82]. In this way, relevant metabolic pathways of *C. variabile *DSM 44702 described below were linked with the transcriptional regulatory network of this cheese isolate.

### Specific features of *C. variabile *DSM 44702 related to carbohydrate metabolism and its regulation in the cheese habitat

**Figure 6:**
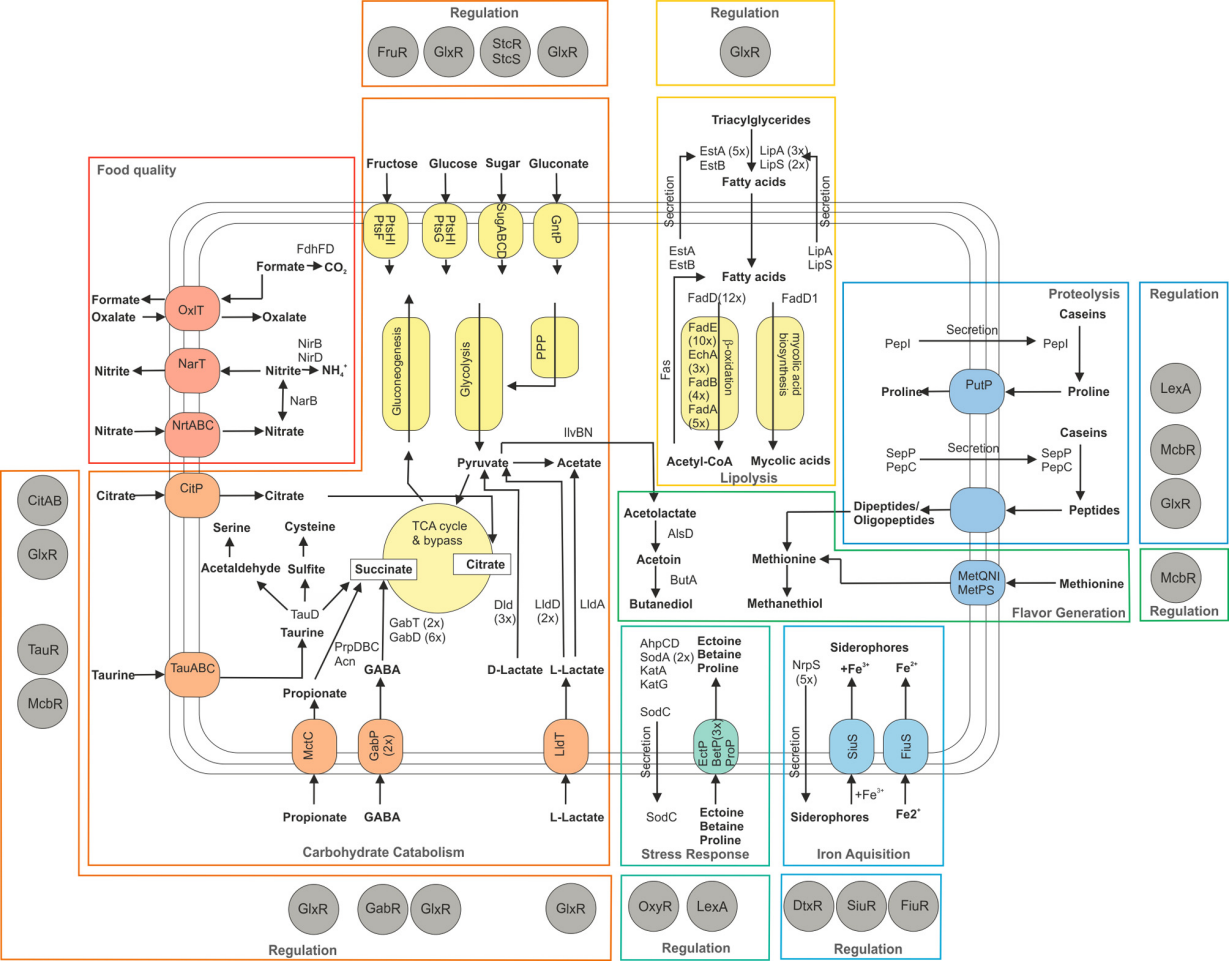
**Metabolic features of *C. variabile *DSM 44702 relevant for the lifestyle on the surface of smear-ripened cheeses and during cheese ripening**. The selected metabolic and regulatory features were deduced from the annotation of the complete genome sequence. Processes and relevant proteins associated with central carbohydrate metabolism, lipolysis and fatty acid metabolism, proteolysis and amino acid uptake, iron acquisition, stress responses, food quality, as well as flavor generation are indicated and labeled. Relevant transport systems are shown within the cell membrane. Numbers in parenthesis indicate the number of paralogs present in the *C. variabile *DSM 44702 genome. Relevant transcription regulators are shown by grey circles and boxed according to the physiological module they play a regulatory role in. Abbreviations: GABA, γ-amino butyric acid; PPP, pentose phosphate pathway; TCA, tricarboxylic acid cycle.

The characteristic features of the central carbohydrate metabolism in *C. variabile *DSM 44702 with respect to the ripening of smear-ripened cheeses are in part specified by genes providing the ability to metabolize citrate, L-lactate, taurine, and γ-amino butyric acid (Figure 6). These metabolites are well-known as prominent components on the surface of smear-ripened cheeses from several previous studies [1,83-86].

#### Citrate uptake in *C. variabile *DSM 44702

The metabolic pathway reconstruction of *C. variabile *DSM 44702 revealed the presence of the *citP *gene encoding a citrate transporter of the CitMHS protein family (Figure 6). Members of this transporter family import citrate in symport with Mg^2+ ^or Ca^2+ ^ions. Citrate is naturally occuring in milk, containing 8 to 9 mM of citrate during fermentation [87]. Therefore, *C. variabile *DSM 44702 can benefit from the natural compositon of the cheese habitat by importing citrate as an additional carbon and energy source. Citrate metabolism is widespread among lactic starter bacteria and can contribute to the synthesis of flavor compounds, such as acetoin and butanediol [88-91].

The expression of the citrate transporter gene *citP *is most likely regulated by the two-component signal transduction system CitAB. The corresponding *citAB *genes are located upstream of *citP *in the *C. variabile *DSM 44702 genome. The orthologous two-component system of *C. glutamicum *ATCC 13032 was shown to regulate genes involved in citrate uptake and metabolism [92]. Target genes of the CitAB system are apparently coregulated by the cAMP-sensing transcription regulator GlxR in this species [74]. The GlxR protein is currently the only known global transcription regulator in corynebacteria, connecting different functional modules of the gene-regulatory network [93]. The *glxR *gene is conserved among all hitherto sequenced corynebacterial species and also present in *C. variabile *DSM 44702. A GlxR binding site was detected upstream of the *citP *coding region, indicating that the expression of this gene is under local control by CitAB and under global control by GlxR in *C. variabile *DSM 44702.

#### Lactate metabolism in *C. variabile *DSM 44702

Two main sources of carbon available to microorganisms at the beginning of cheese ripening are lactose and lactate [1]. Lactose is rapidly converted to lactate in the early days of cheese ripening by the large number of lactic starter bacteria present in the cheese. Lactate is thus the most important carbon source after a few days of cheese ripening [1,94]. According to the taxonomic description and genome sequence data, *C. variabile *DSM 44702 is unable to utilize lactose [4]. On the other hand, the growth rate of *C. variabile *and lactate dehydrogenase activity were significantly increased in the presence of lactate [95]. The *C. variabile *DSM 44702 genome contains the *lldT *gene coding for L-lactate permease involved in L-lactate uptake, which is located upstream of the *lldD1 *gene. A paralog of *lldD1*, named *lldD2*, is located elsewhere on the chromosome. Both genes encode L-lactate dehydrogenase (EC 1.1.1.27) that facilitates the conversion of L-lactate to pyruvate. In addition, L-lactate can be converted to acetate by lactate 2-monooxygenase (EC 1.13.12.4) encoded by the *lldA *gene of *C. variabile *DSM 44702. D-Lactate is also used as substrate by three *dld *paralogs (*dld1*-*dld3*) coding for D-lactate dehydrogenases (EC 1.1.1.28). D-Lactate is a natural product in smear-ripened cheeses formed by lactic acid bacteria that can produce both stereoisomers of lactic acid [94]. Due to efficient metabolism of L-lactate and D-lactate, *C. variabile *can probably grow from the beginning of the ripening process and is independent of significant yeast growth during the initial stages of cheese ripening [17]. GlxR binding sites were detected upstream of the putative *lldT*-*lldD1 *operon, upstream of the *lldD2 *coding region as well as in front of the paralogous *dld *genes (data not shown). Lactate metabolism of *C. variabile *DSM 44702, with the exception of *lldA *expression, is thus under global control by GlxR, as it was demonstrated previously in *C. glutamicum *ATCC 13032 [74,96].

#### Taurine utilization by *C. variabile *DSM 44702

Taurine (2-aminoethanesulfonic acid) is a sulfonated amino acid that is not incorporated into polypeptides. It derives from the metabolism of sulphur-containing amino acids and is the most abundant free amino acid in goat's milk, with a mean value of 66.2 mg/l [97]. The amount of taurine in cow's milk is considerably lower (1.6-10 mg/l) [98]. The *C. variabile *DSM 44702 genome contains a gene cluster involved in the assimilation of taurine (Figure 6). The respective genomic region comprises the *tauCBA *genes encoding a predicted taurine transporter of the ABC superfamily, the *tauD1 *gene encoding a putative taurine dioxygenase, and the *tauR *gene coding for a ROK-type transcription regulator. Moreover, *C. variabile *DSM 44702 contains a paralog of *tauD1 *not located in this gene cluster, *tauD2*, encoding a second taurine dioxygenase. This enzyme (EC 1.14.11.17) catalyzes the key step in taurine assimilation by generating sulfite, aminoacetaldehyde, and succinate (Figure 6). As the resulting sulfite can be converted to cysteine, the cheese component taurine can serve as a combined sulphur and carbon source for *C. variabile *DSM 44702.

The expression of the *tauCBA *and *tauD1 *genes is apparently regulated by TauR, a ROK-type transcription regulator, whose corresponding gene is located upstream of the *tauD1CBA *operon. ROK-type regulators are known from *C. glutamicum *ATCC 13032 to contribute to the transcriptional control of sulphur metabolism that is under hierarchical control by the TetR-type regulator McbR [99-101]. McbR belongs to the core set of corynebacterial transcription regulators [74] and acts as the master regulator of all aspects of sulphur metabolism in *C. glutamicum *and *C. jeikeium *[101,102]. The McbR protein is also encoded in *C. variabile *DSM 44702 by the *mcbR *gene that is located at the boundary of the small duplicated genomic region CVAR_2865-CVAR_2874 (*mcbR*). The second copy of this DNA segment is part of the iron acquisition island II (Figure 3), comprises the coding regions CVAR_2821-CVAR_2830, and ends with a 5' truncated version of the *mcbR *gene, named *mcbR2*. A genome-wide search for McbR binding sites in *C. variabile *DSM 44702 detected 23 operators that are probably controlling 41 genes involved in (*S*-adenosyl)methionine biosynthesis, methionine import, cysteine biosynthesis, and sulfate reduction (data not shown). In addition, McbR binding sites were detected in the *tauR*-*tauD1 *intergenic region and in front of the *tauD2 *coding region. This data strongly indicates that all genes involved in taurine assimilation are embedded in the sulphur regulatory network of *C. variabile *DSM 44702.

#### GABA utilization by *C. variabile *DSM 44702

GABA (γ-amino butyric acid) is a four-carbon, non-proteinogenic amino acid known to occur in cheese with a varying concentration that is strongly affected by the protocol of cheese manufacture, the type of primary starter cultures, the cheese microbiota, and the ripening conditions [85,86]. Several species of lactic starter bacteria are able to synthesize GABA from L-glutamate by glutamate decarboxylase (EC 4.1.1.15) [85] that is widely distributed among eukaryotes and prokaryotes [103] and also encoded in the *C. variabile *DSM 44702 genome by the *gadB *gene. In addition, GABA is degraded to succinate by the enzymes γ-aminobutyrate aminotransferase (EC 2.6.1.19) and succinate semialdehyde dehydrogenase (EC 1.2.1.16) in *C. variable *DSM 44702 (Figure 6). These three enzymatic reactions belong to a metabolic pathway known as GABA shunt [104]. Two genes encoding GABA transporters (*gabP1 *and *gabP2*) were detected in *C. variabile *DSM 44702. The first one is located in a cluster comprising the genes *gabD1 *encoding succinate semialdehyde dehydrogenase, *gabT1 *coding for γ-aminobutyrate aminotransferase, and *gabR1 *specifying a transcription regulator. The second gene cluster attributed to GABA utilization is composed of *gabP2*, *gabT2*, and *gabR2*, and is thus lacking a *gabD *component. In total, six potential *gabD *genes (*gabD1*-*gabD6*) are distributed across the *C. variabile *DSM 44702 chromosome.

According to this genome annotation, the uptake and catabolism of GABA in *C. variabile *DSM 44702 is represented by at least three gene regions (Figure 6). The first gene cluster (*gabT1*-*gabD1*-*gabP1*) is preceded by the regulatory gene *gabR1 *coding for a transcription regulator of the COG2508 class. Likewise, the second gene cluster (*gabT2*-*gabP2*) is preceded by the regulatory gene *gabR2 *also coding for a transcription regulator assigned to the COG2508 class. The presence of this type of regulatory genes upstream of *gab *gene clusters is conserved among GABA-utilizing corynebacteria [105], suggesting that the respective regulators of the COG2508 class are involved in the transcriptional control of the divergently arranged *gabT(D)P *genes. The succinate semialdehyde dehydrogenase gene *gabD2 *is located elsewhere on the chromosome and organized in an operon with the regulatory gene *gabR3*, which encodes a transcription regulator of the LysR protein family [106]. Moreover, GlxR binding sites were detected in the intergenic region of *gabR1*/*gabT1 *and *gabR2*/*gabT2*, as well as upstream of the *gabR3 *coding region, indicating that the respective GABA-related genes of *C. variabile *DSM 44702 are under hierarchical regulation by local GabR regulators and the global cAMP-sensing regulator GlxR. Integration of GABA utilization into the global GlxR network of *C. variabile *DSM 44702 is reasonable, because the central pathway intermediate succinate is finally generated by the GABA shunt (Figure 6). Furthermore, it is remarkable that GABA utilization as well as taurine and propionate metabolism channel succinate into the tricarboxylic acid cycle, as the typical generation of this compound by succinyl-CoA synthetase is not encoded in *C. variabile *DSM 44702.

### Nitrate, nitrite, and oxaloacetate metabolism of *C. variabile *DSM 44702 in the cheese habitat

Low amounts of nitrates and nitrites are natural constituents of many types of cheese [107]. Both compounds may exert important effects on human health when present in higher concentrations, as they may react with naturally present amines to form potent carcinogens. The content of nitrates and nitrites in cheese is therefore strictly regulated by law in many European countries. The annotation of the *C. variabile *DSM 44702 genome sequence revealed the presence of the *nrtABC *genes encoding an ABC-type transport system involved in nitrate uptake (Figure 6). Nitrate can be reduced by a putative nitrate reductase (EC 1.7.99.4) encoded by the *narB *gene of *C. variabile *DSM 44702, which is located upstream of *nirB *and *nirD*, both coding for subunits of nitrite reductase (EC 1.7.1.4). This enzyme converts nitrite to ammonium hydroxide, resulting in the detoxification of nitrite and the generation of a suitable nitrogen source for *C. variabile *DSM 44702. Alternatively, nitrite can be exportet by the putative nitrite extrusion protein NarT, which is encoced in the same gene cluster by the *narT *gene.

Moreover, the oxalate:formate antiporter gene *oxlT *was identified in *C. variabile *DSM 44702 (Figure 6). The OxlT protein is a member of the major facilitator superfamily and exchanges formate for oxalate across the cytoplasmic membrane [108]. Oxalate is present in milk and cheese in relatively small amounts. Nevertheless, oxalic acid can react with calcium ions in the milk producing calcium oxalate, thereby preventing this vital nutrient from being absorbed in the human intestine [109]. In principle, oxalate can be converted to formate by oxalate decarboxylase (EC 4.1.1.2), but the respective *oxdD *gene is absent in the genome of *C. variabile *DSM 44702. However, formate can be oxidized in *C. variabile *DSM 44702 by formate dehydrogenase (EC 1.2.1.2) encoded by the *fdhFD *genes (Additional file 1). Due to import of oxalic acid by *C. variabile *DSM 44702, the binding of calcium ions probably occurs to a lower degree in smear-ripened cheeses in the presence of this strain. Therefore, the gene equipment of *C. variabile *DSM 44702 provides a substantial benefit for the quality of smear-ripened cheeses.

### Lipolysis and fatty acid metabolism by *C. variabile *DSM 44702 in the cheese habitat

Cow's milk contains high amounts of lipids and triacylglycerides (about 35 g/l), which may serve as carbon and energy sources for the microflora of smear-ripened cheeses [110]. Free fatty acids derive from breakdown of triacylglycerides, and their further degradation can lead to volatile compounds having low olfactory thresholds in cheese flavor perception [111]. In smear-ripened cheeses, more than 4 g/kg of free fatty acids are available for the surface microbiota [1]. The genome of *C. variabile *DSM 44702 is equipped with a large set of genes encoding enzymes involved in lipolysis and the degradation of fatty acids (Figure 6). It contains five genes (*estA1*-*estA5*) encoding secreted esterases of the SGNH-hydrolase superfamily and the *estB *gene coding for an esterase of the AB hydrolase superfamily [112]. Secreted lipases are encoded by five genes in the *C. variabile *DSM 44702 genome. The paralogous *lipA *genes (*lipA1*-*lipA3*) encode class 2 lipases that hydrolyse ester bonds in triacylglycerol giving diacylglycerol, monoacylglycerol, glycerol, and free fatty acids [112]. The genes *lipS1 *and *lipS2 *belong to the LIP superfamily containing lipases with broad lipolytic activities [112]. All these enzymes may thus contribute to the generation of free fatty acids from precursor molecules present in the cheese habitat.

Furthermore, 13 *fadD *genes encoding acyl-CoA synthetases were identified in the genome of *C. variabile *DSM 44702, including the *fadD1 *gene that is involved in mycolic acid biosynthesis [113]. The amino acid sequences of the FadD enzymes in *C. variabile *DSM 44702 revealed low levels of overall similarity, but the characteristic AMP-binding motif of acyl-CoA synthetase is highly conserved among the twelve paralogs (FadD2-FadD13). In principle, acyl-CoA synthetases are involved in activating free fatty acids to form acyl-CoA of various chain lengths concomitant with the transport into the bacterial cell [114]. The amino acid sequence diversity of the predicted acyl-CoA synthetases might indicate different substrate specificities of these enzymes, which would enable *C. variabile *to utilize a broader range of fatty acid substrates in its natural cheese habitat, probably leading to a growth advantage as a well-adapted species.

In addition to the set of *fadD *genes, a complete β-oxidation pathway for fatty acid degradation is encoded in the *C. variabile *DSM 44702 genome (Figure 6). In this pathway, the acyl-CoA is converted into a product with a shortened acyl chain (n-2) and acetyl-CoA that can be used in the tricarboxylic acid cycle to generate energy, whereas the shortened acyl-CoA is used in a new round of β-oxidation [115]. In principle, *C. variabile *DSM 44702 can utilize exogenous fatty acids also as carbon sources, since the glyoxylate bypass genes *aceA *and *aceB *are present and the gluconeogenesis pathway is complete (Additional file 1). The predicted enzymes involved in β-oxidation of *C. variabile *DSM 44702 are encoded by ten paralogs of *fadE *(encoding acyl-CoA dehydrogenase), the bifunctional *fadB1 *gene (enoyl-CoA hydratase/hydroxyacyl-CoA dehydrogenase), three monofunctional *fadB *genes (hydroxyacyl-CoA dehydrogenase), three *echA *genes (enoyl-CoA hydratase), and five paralogs of *fadA *(ketoacyl-CoA thiolase). The amino acid sequences of these paralogous gene families vary substantially, again suggesting diverse substrate specificities of the respective enzymes, at least in the case of the ten acyl-CoA dehydrogenases. Interestingly, all genes coding for secreted esterases or lipases and almost all *fad *and *echA *genes of *C. variabile *DSM 44702 contain at least one GlxR binding site in their upstream regions (data not shown), indicating that the complete metabolic process of lipolysis and β-oxidation is under global control by the GlxR protein.

In contrast to the lipid-auxotrophic relatives *C. jeikeium *and *C. urealyticum*, the metabolic pathway analysis of the *C. variabile *DSM 44702 genome revealed the presence of the fatty acid synthase gene *fas*, showing that growth of this strain is not dependent on the availability of exogenous fatty acids [31,34]. These compounds are, moreover, building blocks for the synthesis of corynomycolic acids, which are major constituents of the corynebacterial cell envelope [116]. It was shown previously that a polyketide synthase encoded by the *pks13 *gene represents a condensase that catalyzes the key step in the biosynthesis of corynomycolic acids, in conjunction with an acyl-CoA carboxylase and a distinct acyl-AMP ligase [117-119]. The *pks13 *gene is located in a conserved cluster in the *C. variabile *DSM 44702 genome, including genes coding for an acyl-CoA carboxylase (*accD3*), an acyl-CoA synthetase/acyl-AMP ligase (*fadD1*), the envelope lipids regulation factor ElrF (*elrF*), and trehalose corynomycol transferases (*cmt*). It is noteworthy that eight genes encoding trehalose corynomycolyl transferases (*cmtA*-*cmtH*) were identified in the *C. variabile *DSM 44702 genome, catalyzing the transfer of mycolic acids from trehalose monocorynomycolate to another molecule of trehalose monocorynomycolate or on the cell wall arabinogalactan [116]. Five of these genes (*cmtC*-*cmtG*) are clustered with the *pks13 *gene.

A second gene cluster involved in fatty acid metabolism of *C. variabile *DSM 44702 includes genes coding for putative acyl-CoA carboxylase subunits (CVAR_2092 and CVAR_2091), an acyl-CoA dehydrogenase domain-containing protein (CVAR_2090), an enoyl-CoA hydratase domain-containing protein (CVAR_2089), a citrate lyase β-subunit (*citE*), an acyl-CoA synthetase (*fadD6*), aycl-CoA:3-ketoacid-CoA transferase subunits (*scoA *and *scoB*), and a ketoacyl-CoA thiolase (*fadA4*). A similar gene cluster is present only in the genome of the lipophilic isolate *C. jeikeium *K411, whereas some genes were found in a similar genetic arrangement in the genome of *Mycobacterium tuberculosis *H37Rv [120]. As most of the genes present in these conserved clusters are linked to fatty acid degradation, they might be involved in the activation and subsequent degradation of a distinct type of fatty acid substrate, whose chemical composition is currently unknown.

### Proteolysis and utilization of caseins by *C. variabile *DSM 44702 in the cheese habitat

Protein degradation contributes to cheese texture and the generation of cheese flavor in all varieties of smear-ripened cheeses, as many relevant flavor compounds derive directly from amino acids [1,87,91,121]. Milk and especially cheese have a high protein content, which is represented by a mixture of αS1-, αS2-, and β-caseins [1]. Extracellular caseins can be degraded by *C. variabile *DSM 44702 by means of secreted proteolytic enzymes, such as a secretory serine protease encoded by the *sepP *gene and a secretory aminopeptidase encoded by the *pepC *gene (Figure 6). In addition, amino-terminal prolines are released from peptides by the enzymatic action of proline iminopeptidase (EC 3.4.11.5) encoded by the *pepI *gene. The enzymatic activity of an extracellular proline iminopeptidase from *C. variabile *NCDO 2101 was demonstrated previously [122]. Since caseins are very rich in proline residues and proline is the most abundant amino acid in cheese, it may constitute a prominent substrate for *C. variabile*. The import of "free" or released proline is facilitated in *C. variabile *DSM 44702 by the amino acid transporters ProP and PutP (Additional file 3). The subsequent oxidation of proline to glutamate is catalyzed as a two-step reaction by a bifunctional proline dehydrogenase (EC 1.5.99.8 and EC 1.5.1.12) encoded by the *putA *gene [123].

Interestingly, McbR binding sites were detected in the upstream regions of *sepP *and *pepC*, suggesting the integration of these genes into the sulphur regulatory network of *C. variabile *DSM 44702. Therefore, proteolysis might be used by *C. variabile *DSM 44702 to cover the demand for sulphur-containing amino acids and is probably enhanced under sulphate starvation conditions. Other genes assigned to proteolysis by *C. variabile *DSM 44702 are also linked to unfavorable environmental conditions by regulatory features. The proline importer gene *putP*, for instance, is specified by the presence of a typical SOS box and seems to be under the direct transcriptional control by the SOS response regulator LexA, as previously demonstrated in *C. glutamicum *[124]. Moreover, the proline iminopeptidase gene *pepI *is organized in an operon and located downstream of *sodC *in the *C. variabile *DSM 44702 genome. The *sodC *gene encodes a secreted copper, zinc-dependent superoxide dismutase (EC 1.15.1.1) that is characterized by a lipobox motif to be anchored in the cell membrane [64]. The extracellular location of SodC indicates that it helps to protect the cell surface of *C. variabile *against superoxide generated externally by the microbiota of smear-ripened cheeses. The gene arrangement of *pepI *and *sodC *implies that the proline iminopeptidase gene plays a role in the responses to external stresses, which is reasonable since proline can be used as compatible solute in corynebacteria under osmotic stress conditions [125].

### Osmotic and oxidative stress responses of *C. variabile *DSM 44702

Due to permanently changing environmental conditions during the ripening process of smear-ripened cheeses, the bacteria of the cheese microbiota are constantly exposed to several external stresses, such as pH stress and osmotic stress [110,126]. As the pH of the cheese curd can be as low as 4.5 during the early stage of cheese processing, *C. variabile *has to cope with this external stress condition. It was demonstrated in *C. glutamicum *that potassium transport is essential for corynebacterial pH homeostasis and growth at acidic pH [127]. The potassium channel CglK was found to be relevant for the maintenance of the internal pH and the adjustment of the membrane potential. The genome sequence of *C. variabile *DSM 44702 was therefore screened for the presence of genes coding for actinobacterial potassium transporters, of which seven different types were described very recently [128]. *C. variabile *DSM 44702 encodes a homolog of the potassium channel CglK (CVAR_2147) and a Kef-type potassium transport system (CVAR_0647). Moreover, a sodium or potassium/H+ antiporter of the NhaP-type (CVAR_1814) might contribute to pH homeostasis in *C. variabile *DSM 44702.

*C. variabile *DSM 44702 is also well equipped with genes offering protection from osmotic stress that derives from high salt concentrations on the cheese surface during the ripening process (Figure 6). One mechanism to overcome this osmotic stress is the accumulation of osmoprotectants, such as ectoine, proline, and glycine betaine in the cytoplasm [125]. The genome of *C. variabile *DSM 44702 contains the *ectP *gene encoding an ectoine transporter, *proP *coding for an osmoregulated proline transporter, and six genes (*betP1*-*betP3 *and *betT1*-*betT3*) encoding putative betaine/carnitine/choline transporters of the BCCT family. Betaine can be synthesized in two steps from the precursor choline by choline oxidase (EC 1.1.3.17) and betaine-aldehyde dehydrogenase (EC 1.2.1.8) mediated reactions. Milk and cheese contain high amounts of choline [129], making it available as precursor for the synthesis of betaine by *C. variabile*. The *C. variabile *DSM 44702 genome contains two *betA *genes (*betA1 *and *betA2*) encoding choline oxidases, of which the *betA1 *gene is clustered with the betaine-aldehyde dehydrogenase gene *betB *and the putative choline transporter gene *betT1*. The homologous uptake systems for osmoprotectants were extensively studied in *C. glutamicum *ATCC 13032 [130], as was the two-component system MtrAB that is involved in the transcriptional control of the genes encoding osmoregulated compatible solute carriers [131]. The *mtrAB *gene pair was also identified in the genome of *C. variabile *DSM 44702, suggesting the presence of a similiar MtrAB-controlled network of osmoprotection in this species.

The *C. variabile *DSM 44702 genome is moreover equipped with several genes involved in oxidative stress responses, such as two genes (*katA *and *katG*) encoding catalases (EC 1.11.1.6), duplicated genes (*sodA1 *and *sodA2*) coding for intracellular manganese-dependent superoxide dismutases (EC 1.15.1.1), and the aforementioned secreted copper, zinc-dependent superoxide dismutase gene *sodC*. Additional functions involved in oxidative stress responses are provided by *bcp *coding for a peroxiredoxin, *tpx *encoding a thiol peroxidase, and the *ahpCD *gene pair encoding the subunits of alkyl hydroperoxide reductase (EC 1.11.1.15). The latter protein is an antioxidant enzyme responsible for directly reducing organic hyperoxides [132], whereas thiol peroxidase represents an oxidative stress defense system that uses reduction equivalents from thioredoxin and thioredoxin reductase to reduce alkyl hydroperoxides [133]. Thioredoxin (EC 1.8.1.8) and thioredoxin reductase (EC 1.8.1.9) are also encoded by the *trxA *and *trxB *genes in the genome of *C. variabile *DSM 44702. The Bcp protein belongs to the bacterioferritin comigratory protein subfamily of the peroxiredoxin superfamily and represents also a thioredoxin-dependent thiol peroxidase [112].

Furthermore, the *C*. *variabile *DSM 44702 genome contains the regulatory gene *oxyR *encoding a homolog of the central regulator of oxidative stress responses in bacteria [134,135]. The *oxyR *gene belongs to the core set of corynebacterial transcription regulators [74] and is located adjacent to the *ahpCD *gene pair of *C. variabile *DSM 44702. This gene order is also conserved in several corynebacterial genomes and in genomes of slow-growing mycobacteria, where *oxyR *is involved in the activation of *ahpCD *gene expression [136,137]. According to this conserved gene arrangement in actinobacterial species and experimental data from mycobacteria, it is likely that OxyR controls at least the expression of the *ahpCD *genes in *C. variabile *DSM 44702. The *katG *gene of *C. variabile *DSM 44702 is probably under transcriptional control by LexA, as a DNA-binding site (SOS box) of this regulator was detected in front of the *katG *coding region (data not shown). The LexA protein is the key component of the bacterial SOS response [138], and the *lexA *gene is conserved in all corynebacterial genomes [74].

### Iron acquisition by *C. variabile *DSM 44702 in the iron-depleted cheese habitat

For most bacteria, iron is essential as a cofactor for proteins involved in important cellular functions, such as DNA biosynthesis and aerobic respiration [139]. Thus, iron acquisition is a vital function for bacterial survival. However, milk and cheese contain the iron-binding protein lactoferrin that can deny iron to microorganisms in such a way that smear-ripened cheeses contain only small amounts of readily available iron [140]. To circumvent this limitation of iron in the cheese habitat, bacteria of the cheese microflora have evolved several molecular strategies, including the synthesis of siderophores during the ripening process [141]. Siderophores are high-affinity iron-chelating compounds, often synthesized by non-ribosomal peptide synthetases in response to low-iron availability in the environment [142]. The genome of *C. variabile *DSM 44702 contains five genes (*nrpS1*-*nrpS5*) encoding proteins with sequence similarities to multi-domain peptide synthetases (Figure 6). The peptide synthetase genes *nrpS2 *to *nrpS5 *are clustered in iron-acquisition island II of the *C. variabile *genome (Figure 3). This genomic island contains also the *entC2 *and *entE2 *genes, coding for isochorismate synthase (EC 5.4.4.2) and 2, 3-dihydroxybenzoate-AMP ligase (EC 2.7.7.58), respectively. These gene products revealed similarities to proteins involved in the biosynthesis of the catechol siderophores bacillibactin from *Bacillus subtilis *[143] and enterobactin from *Escherichia coli *[144]. Additional enzymatic functions involved in the biosynthesis of catechol siderophores are encoded in the *entA*-*entC1*-*entB*-*fes *gene cluster of *C. variabile *DSM 44702. The *entA *gene encodes 2, 3-dihydro-2, 3-dihydroxybenzoate dehydrogenase (EC 1.3.1.28) and the *entB *gene codes for isochorismatase (EC 3.3.2.1). The product of the *fes *gene is similar to enterobactin esterase that is probably required for the removal of iron from the siderophore. In conjunction with a non-ribosomal peptide synthetase, these genes provide a complete pathway for the synthesis of catechol siderophores with chorismate as the precursor [145]. Among the cluster 3 species of the genus *Corynebacterium*, similar siderophore biosynthesis genes are only present in the genome of *C. jeikeium *K411 [34].

**Figure 7:**
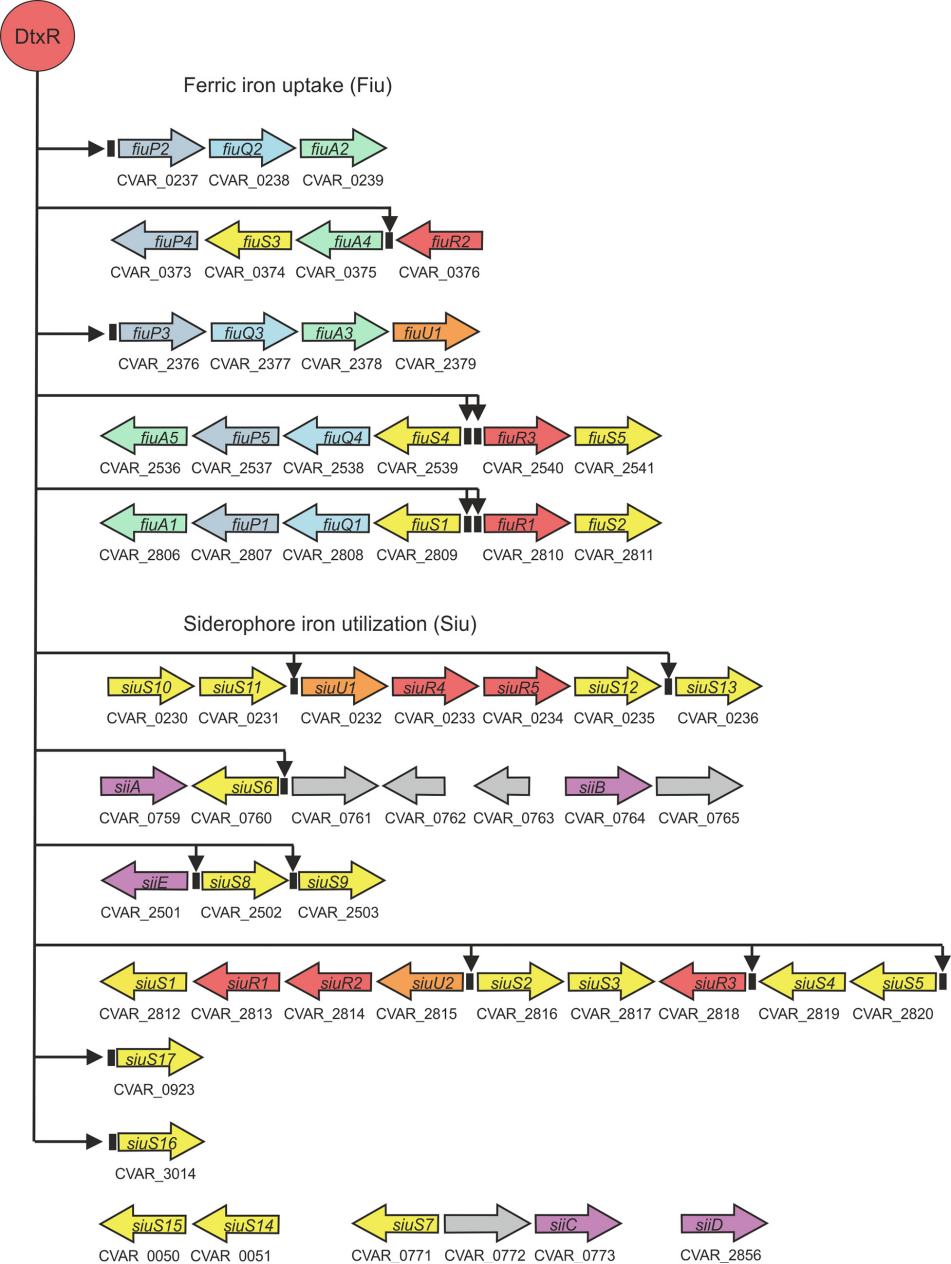
**Gene regions associated with iron acquisition in *C. variabile *DSM 44702**. Gene clusters probably involved in the uptake of ferric iron (*fiu*) and iron-siderophores (*siu*) are presented. Detected DtxR binding sites are indicated as black boxes upstream of the coding regions. Genes encoding iron uptake and utilization systems are colored according to their functional assignments, whereas grey arrows indicate genes of unknown functions present in the *siu *clusters.

Further analysis of the *C. variabile *DSM 44702 genome sequence revealed the presence of 29 genes with predicted functions in the utilization of iron-siderophores, named either *siu *(siderophore-iron utilization protein) or *sii *(siderophore-iron interacting protein) (Figure 7). *Siu *gene clusters are represented by *siuS *genes encoding substrate-binding proteins (*siuS1*-*siuS17*), *siuU *genes encoding siderophore utilization proteins (*siuU1 *and *siuU2*), and *sii *genes coding for siderophore-interacting proteins (*siiA*-*siiE*), which might be necessary for the removal of iron from iron-siderophore complexes [110]. In addition, five regulatory genes (*siuR1*-*siuR5*) encoding transcription regulators of the AraC protein family were assigned to these gene clusters. Interestingly, numerous genes involved in iron utilization are located in the iron acquisition islands I (CVAR_230-CVAR_0239) and II (CVAR_2806-CVAR_2820) of the *C. variabile *genome (Figure 3).

A further aspect of iron aquisition is the transport of this trace element into the bacterial cell. The *C. variabile *DSM 44702 genome contains 23 genes with assigned functions in ferric iron uptake (*fiu*) (Figure 7). The *fiu *gene clusters are represented by genes coding for substrate-binding proteins (*fiuS*), permease components (*fiuP *and *fiuQ*), ATP-binding proteins (*fiuA*), and an iron-utilization protein (*fiuU1*). In addition, three *fiu *gene clusters contain regulatory genes (*fiuR1*-*fiuR3*) encoding transcription regulators of the AraC family (Figure 7). The accumulation of genes coding for AraC-like proteins in the *siu *and *fiu *gene clusters explains the large number of transcription regulators assigned to this protein family in *C. variabile *DSM 44702 (Figure 5), when compared with the regulatory repertoires in other corynebacterial genomes [78,80,81].

As iron limitation poses a problem for the microflora of the cheese surface, the microorganisms have to compete for iron, but nonetheless, they must regulate iron metabolism to prevent excess iron that can initiate the generation of toxic oxygen radicals from normal products of metabolism by the Fenton reaction [146]. Bacteria have solved the problem of iron acquisition and homeostasis by encoding uptake systems that are tightly regulated at the transcriptional level [139]. DtxR is the key transcription regulator controlling complex gene-regulatory networks involved in iron homeostasis in corynebacteria [74,147]. The complete genome sequence of *C. variabile *DSM 44702 was screened for the presence of DtxR binding sites, using the experimentally validated binding motif from *C. glutamicum *ATCC 13032 as input [147]. This genome-wide motif search detected 26 operators that are probably controlling the expression of 62 target genes, including for instance *dps *(DNA protection during starvation protein), *ftn *(ferritin), *hmuO *(heme oxygenase), *sdhCAB *(succinate dehydrogenase), and the *suf *operon involved in Fe-S cluster assembly. Among the detected operators, 17 are located in the *siu *and *fiu *gene clusters of *C. variabile *DSM 44702, regulating in total 41 genes that are organized in 18 transcription units (Figure 7). This result indicates that almost all *siu *and *fiu *gene clusters are integrated into the iron-regulatory network of *C. variabile *DSM 44702 that obviously exerts a hierarchical control of gene expression by DtxR in concert with the AraC-type regulators of these clusters. Such a complex transcriptional regulation of iron homeostasis has not been detected in any sequenced corynebacterial genome before [74].

### Contribution of *C. variabile *DSM 44702 to flavor generation during cheese ripening

The flavor compounds commonly found in smear-ripened cheeses are produced by the cheese microbiota as a diverse group of volatile substances, including volatile sulphur-containing compounds, esters, aldehydes, and ketones [148]. Half of these compounds derive from lactose fermentation, citrate degradation, and a few from lipolysis. The second half derives from amino acid catabolism by complex metabolic pathways [87]. Methanethiol, for instance, derives from methionine and is a prominent aroma compound in smear-ripened cheeses. Several cheese-related strains of *B. linens *produce high amounts of methanethiol due to the presence of L-methionine-γ-lyase (EC 4.4.1.11) [149]. This enzyme converts methionine to methanethiol via a γ-elimination step. A corresponding *mgl *gene was not detectable in the genome of *C. variabile *DSM 44702, although this strain was shown previously to produce methanethiol [4]. This observation indicates that other enzymes must be responsible for the synthesis of methanethiol in *C. variabile *DSM 44702. Cystathionine β-lyase (EC 4.4.1.8) and cystathionine γ-lyase (EC 4.4.1.1) catalyze similar reactions in lactococci [150,151], but only the former enzyme is encoded by the *aecD *gene in *C. variabile *DSM 44702.

Acetoin (3-hydroxybutanone) is another aroma compound of smear-ripened cheeses and gives butter its characteristic flavor [87,152]. *C. variabile *DSM 44702 produces acetoin from pyruvate, with acetolactate being an intermediate. The conversion of acetolactate to acetoin is mediated by acetolactate decarboxylase (EC 4.1.1.5) encoded by the *alsD *gene (Figure 6). Moreover, acetoin can be converted to 2, 3-butanediol by the enzymatic activity of acetoin reductase (EC 1.1.1.4) encoded by the *butA *gene in *C. variabile *DSM 44702. Butanediol is also an important flavor compound in some varieties of smear-ripened cheeses [153].

## Discussion

Cheese is one of the oldest dairy products produced by humanity and its origin predates recorded history [1]. Nowadays, hundreds of cheese varieties are produced throughout the world in wide-ranging textures, forms, and flavors. The production processes of all cheese varieties share one common characteristic: the microflora present in each cheese contributes to the typical properties of the final product, such as taste, aroma, and color [1,94]. The complex interactions of these microorganisms and the various biochemical processes occuring during cheese ripening are the result of centuries of adaptation of yeasts, moulds, and bacteria to the cheese habitat [94]. During the traditional ripening of smear-ripened cheeses, the surface flora of old, ripenend cheeses was transfered to young, unripened cheeses by a brining step in the early stage of ripening, a process named "old-young smearing" [154]. Therefore, the combined metabolic activities of a highly adapted bacterial community on the surface of smear-ripened cheeses have evolved over many years to determine all aspects of the mature product [94]. However, the process of "old-young smearing" involves the transfer of a complex, undefined, and variable microflora. Therefore, a significant improvement in the industrial production of smear-ripened cheeses is nowadays achieved by using defined surface starter cultures. *C. variabile *was shown previously to be an important bacterial component on the surface of the smear-ripened cheeses Brick and Gubbeen [4,10,17,20]. Likewise, *C. casei *is also a dominant component on the surface of Gubbeen, but is moreover found to be dominant on the surface of other smear-ripened cheeses, such as Ardrahan, Durrus, and Milleens [11]. Therefore, we reconstructed in this study the metabolic capabilities of *C. variabile *DSM 44702 from the complete genome sequence to understand its adaptation to the cheese surface habitat and its physiological role in cheese ripening and flavor generation.

The *C. variabile *genome (3.43 Mb) contains large regions with high levels of synteny with the chromosome of the close phylogenetic relative *C. jeikeium *(2.46 Mb), a multi-drug resistant nosocomial pathogen [155]. However, the size of the *C. variabile *genome is rather characteristic of environmental corynebacteria [15], such as *C. glutamicum *(3.28-3.31 Mb), *Corynebacterium efficiens *(3.14 Mb), and *Corynebacterium nuruki *(3.11 Mb), than of pathogenic relatives of the cluster 3 subline [31,34,80]. In contrast to the genetic repertoires of the cluster 3 species *C. jeikeium *and *C. urealyticum *that both show a clear reduction of genes associated with catabolic activities of carbon sources, the metabolic reconstruction of *C. variabile *revealed the presence of a complete set of enzymes required for central carbohydrate metabolism, vitamin and cofactor biosynthesis, and the synthesis of amino acids. *C. variabile *is equipped for instance with several additional enzymes and proteins required for the utilization of substrates typically present on the cheese surface, including citrate, lactate, propionate, taurine, and γ-amino butyric acid (GABA). The utilization of the sulfonated amino acid taurine as a combined carbon and sulphur source by *C. variabile *has not been reported so far in conjunction with other bacterial species associated with smear-ripened cheeses. GABA was shown to be synthesized by lactic starter bacteria within the body of 22 Italian cheese varieties, such as Gorgonzola, Mozzarella, and Pecorino [85], and is also utilized as carbon source by *Arthrobacter arilaitensis *[110], which is one of the major bacterial species detected on the surface of smear-ripened cheeses. This bacterium is also able to utilize the stereoisomers L-lactate and D-lactate, but not citrate, presumably as a result of a mutation in the gene cluster encoding the two-component system CitAB necessary for activating the expression of the cognate citrate import system [110].

*C. variabile *is also equipped with an extensive set of enzymes participating in β-oxidation or the degradation of lipids and with enzymes involved in proteolysis. Cheese has a high content of lipids and triacylglycerides [1] that are probably made available for the metabolism of *C. variabile *by the enzymatic actions of secreted lipases and esterases. Likewise, caseins are the major protein sources in milk and cheese [1] and are thus prominent substrates for proteolysis by *C. variabile*, presumably carried out by secreted proteases. Moreover, proline iminopeptidase can contribute to proteolysis [122], as prolines are the major amino acid constituents of caseins [1]. It is obvious that these metabolic specificities of *C. variabile *are closely linked to its niche adaption. Another specific feature of *C. variabile *is its high salt tolerance [4]. The bodies of smear-ripened cheeses are brined with salt solutions prior to the start of the ripening period to control microbial growth and to extend shelf-life [94]. The efficient accumulation of osmoprotectants by *C. variabile *may contribute to its salt tolerance, as a large number of genes accounting for the accumulation of compatible solutes were detected in the genome sequence.

Moreover, the *C. variabile *genome is well equipped with genes involved in iron acquisition, presumably as a result of adaptation to the iron-depleted cheese habitat. At least one complete siderophore biosynthesis cluster was identified in this species, which shows similarity to gene clusters involved in the synthesis of catechol siderophores in *B. subtilis *[143] and *E. coli *[144]. The biosynthesis of siderophores was also observed in cheese-related strains of *B. linens *and *A. arilaitensis *that are frequently found on the surface of smear-ripened cheeses [110,156]. Moreover, about 50 additional genes were assigned to iron-siderophore and ferric iron uptake components, which have few orthologs in other corynebacteria, indicating that the acquisition of iron is a key factor for growth of *C. variabile *on the surface of smear-ripened cheeses. It would be interesting to investigate whether *C. variable *is able to use siderophores produced by other species sharing its habitat, as it has been assumed for *A. arilaitensis *[110].

In principle, flavor compounds in cheese can derive from pathways of lipolysis or proteolysis and the metabolism of lactate and citrate [87]. Volatile sulphur compounds, such as methanethiol, are particularly important in the flavor of smear-ripened cheeses and produced by the bacterial microflora [94]. There is experimental evidence that *C. variabile *produces methanethiol [4], although no methionine-γ-lyase encoding gene (*mgl*) was detectable in this study. Another flavor compound produced by *C. variabile *from pyruvate is acetoin, which is also generated by lactococci in fresh unripened cheese and can be reduced to 2, 3-butanediol by the action of the enzyme acetoin reductase [157]. The corresponding *butA *gene was detected in the *C. variabile *genome, indicating that the synthesis of 2, 3-butanediol might contribute to flavor generation of smear-ripened cheeses. Moreover, lipolysis plays an important role in the formation of cheese flavor in several cheese varieties, such as Swiss cheese [158], Hispánico cheese [159], Domiati cheese [160], and smear-ripened cheeses [87]. The quantification of the production of flavour compounds from fat during cheese ripening by the action of lipases and esterases indicated that lipolysis can reach up 20% in mould-ripened cheeses, such as Camembert [111]. Due to the presence of several lipases and esterases encoded by *C. variabile*, it is likely that this species may also contribute to cheese flavor by producing free fatty acids from lipids and triacylglycerides and by generating volatile compounds with low olfactory threshold values in cheese flavor perception.

Furthermore, the detected regulatory pattern for relevant metabolic pathways led to the conclusion that almost all major processes occuring in *C. variabile *are regulated in a hierarchical manner. We identified local regulators, whose corresponding genes are clustered with their proposed target genes, for instance the *tauR *gene that is located adjacent to the *tauDCBA *cluster involved in uptake and assimilation of taurine, *gabR *genes located upstream of *gabT(D)P *genes responsible for GABA utilization, as well as *siuR *and *fiuR *genes that are embedded in iron-siderophore and ferric iron uptake gene clusters and are all encoding members of the AraC family of transcription regulators. In addition, DNA-binding sites of master regulators known from *C. glutamicum *were detected in the *C. variabile *genome sequence. McbR binding sites, for instance, indicate the connection of the *tauABCD *genes to the sulphur regulon of *C. variabile*, whereas DtxR binding sites within the *siu *and *fiu *gene clusters indicate the integration of the respective genes into an iron-dependent gene-regulatory network. The presence of GlxR binding sites support the view of a hierarchical regulation of several prominent aspects of the *C. variabile *metabolism, including the utilization of γ-amino butyric acid, lipolysis, and β-oxidation.

## Conclusions

The annotation of the *C. variabile *DSM 44702 genome in conjunction with comparative analysis provides evidence that a strong selective pressure exists on the surface of smear-ripened cheeses, concerning the ability to catabolize substrates, such as γ-amino butyric acid, lactate, lipids, and amino acids (mainly proline), and the presence of efficient iron acquisition and salt-tolerance systems. Our analysis of the genome data revealed that *C. variabile *is strongly adapted to this habitat and contributes to the main aspects of cheese quality, such as flavor formation. Future studies will certainly benefit from the comprehensive genomic information on *C. variabile *by integrating this knowledge into models describing the complex cheese ecosystem, which is composed of various bacterial species and yeasts and their microbial interactions.

## Methods

### Bacterial strain and growth conditions

*C. variabile *DSM 44702 (CIP 107183; JCM 12073; LMG S-19265; NCIMB 30131) was originally isolated from the surface of an Irish farmhouse smear-ripened cheese [4]. The strain used in this study was obtained as a lyophilized culture from the German Culture Collection DSMZ (Braunschweig, Germany). *C. variabile *DSM 44702 was formerly described as the type strain of the species *C. mooreparkense*, which turned out to be a later heterotypic synonym for *C. variabile *[4,20]. *C. variabile *DSM 44702 was routinely cultivated in BYT complex medium [161] at 30°C.

### Sequencing of the *C. variabile *DSM 44702 genome

Genomic DNA was purified from *C. variabile *cells by an alkaline lysis procedure [162], using 20-ml aliquots of an overnight culture grown in liquid BYT complex medium that was supplemented with 1.25% (w/v) glycine. The alkaline lysis protocol applied in this study includes two minor modifications: (i) The *C. variabile *cells were incubated in a 30 mg/ml lysozyme solution at 37°C for 1 h. (ii) The harvested *C. variabile *cells were lysed in 0.7 ml 10% (w/v) SDS solution at 37°C for 15 min. A total amount of 5 μg of purified genomic DNA was used for constructing a single-stranded template DNA library. The DNA concentration of the library was measured by using the Agilent RNA 6000 Nano Kit. The preparation and sequencing of the DNA library were performed according to standard protocols from Roche Applied Science. Pyrosequencing of the genomic DNA sample was carried out with the Genome Sequencer FLX System and Titanium chemistry (Roche Applied Science). The sequence reads were assembled with the GS Assembler Software (version 2.3).

### Gap closure of the *C. variabile *DSM 44702 genome

The gap closure was supported by the related reference contig arrangement tool r2cat, using the complete *C. jeikeium *K411 genome sequence as a reference [21]. The remaining gaps in the genome sequence of *C. variabile *were closed by PCR with Phusion hot start high-fidelity DNA polymerase (Finnzymes) and genomic template DNA. All primers used in this study were synthesized by Metabion. The PCR assays were carried out with a TProfessional PCR thermocylcer (Biometra) according to standard protocols (Finnzymes). Amplified DNA fragments linking individual contigs were sequenced by IIT Biotech. All DNA sequences were uploaded into the Consed program [22] and manually inspected to generate the complete genome sequence of *C. variabile *DSM 44702.

### Annotation and bioinformatic analysis of the *C. variabile *DSM 44702 genome

The assembled genome sequence of *C. variabile *was uploaded into the bacterial genome annotation system GenDB [23]. The annotation of the *C. variabile *genome sequence was performed as described previously [34], followed by manual curation. A combined gene prediction strategy was executed by using GLIMMER 2.1 and the CRITICA program suite [163] in conjuction with postprocessing by the RBSfinder tool [164]. Additional regional information on protein-coding regions was obtained with REGANOR [165] and integrated into the final genome annotation. The deduced proteins of *C. variabile *were functionally characterized by automated searches in public databases according to default search parameters of the GenDB system [23]. The origin of chromosomal replication of *C. variabile *was predicted with the web version of the Ori-Finder [25]. The genome plot of *C. variabile *was generated with the DNAPlotter tool [166]. Comparative analyses of the predicted gene content and reconstructions of metabolic properties of *C. variabile *were accomplished by the computer programs EDGAR [30] and CARMEN [167]. The software tool CARMEN supports the *in silico *reconstruction and visualization of automatically derived metabolic networks based on pathway information from the KEGG database and user-defined SBML templates. The visualization and manual curation of pathway maps was performed with the CellDesigner software (version 3.2) [168]. The synteny between the genomes of *C. variabile *DSM 44702 and *C. jeikeium *K411 was calculated with reciprocal best BLASTP matches by the EDGAR software [30]. The detection and classification of the transcriptional regulatory repertoire of *C. variabile *was performed as described previously [76]. The DNA motif discovery for regulon predictions followed a combined workflow, using both position weight matrices and hidden Markov models [96]. The transcriptional regulatory network transfer between corynebacteria on the genome-scale was described in detail previously [82].

The annotated genome sequence of *C. variabile *DSM 44702 has been deposited in the GenBank database with accession number CP002917 and is also available from the RefSeq database with accession number NC_015859.

## Authors' contributions

JS sequenced and annotated the *C. variabile *genome and prepared the manuscript. IM participated in the gap closure process. ET and AT participated in data evaluation. AT supervised the project. All authors read and approved the final version of the manuscript.

## Supplementary Material

Additional file 1 **Pathways involved in the central metabolism of *C. variabile *DSM 44702**. The PDF contains a reconstructed pathway map of the central carbohydrate metabolism. Click here for file

Additional file 2 **Pathways involved in the biosynthesis of vitamins and cofactors by *C. variabile *DSM 44702**. The PDF contains a reconstructed pathway map of vitamin and cofactor biosynthesis. Click here for file

Additional file 3 **Pathways involved in amino acid metabolism of *C. variabile *DSM 44702**. The PDF contains a reconstructed pathway map of amino acid biosynthesis and amino acid transport. Click here for file
