# Supplementary material for: Complete genome sequence of Corynebacterium variabile DSM 44702 isolated from the surface of smear-ripened cheeses and insights into cheese ripening and flavor generation
Source: BMC Genomics. 2011 Nov 3;12:545. doi: 10.1186/1471-2164-12-545 (PMC3219685; doi:10.1186/1471-2164-12-545)
Supplement: Additional file 1 — Pathways involved in the central metabolism of C. variabile DSM 44702. The PDF contains a reconstructed pathway map of the central carbohydrate metabolism. [file 1471-2164-12-545-S1.PDF]

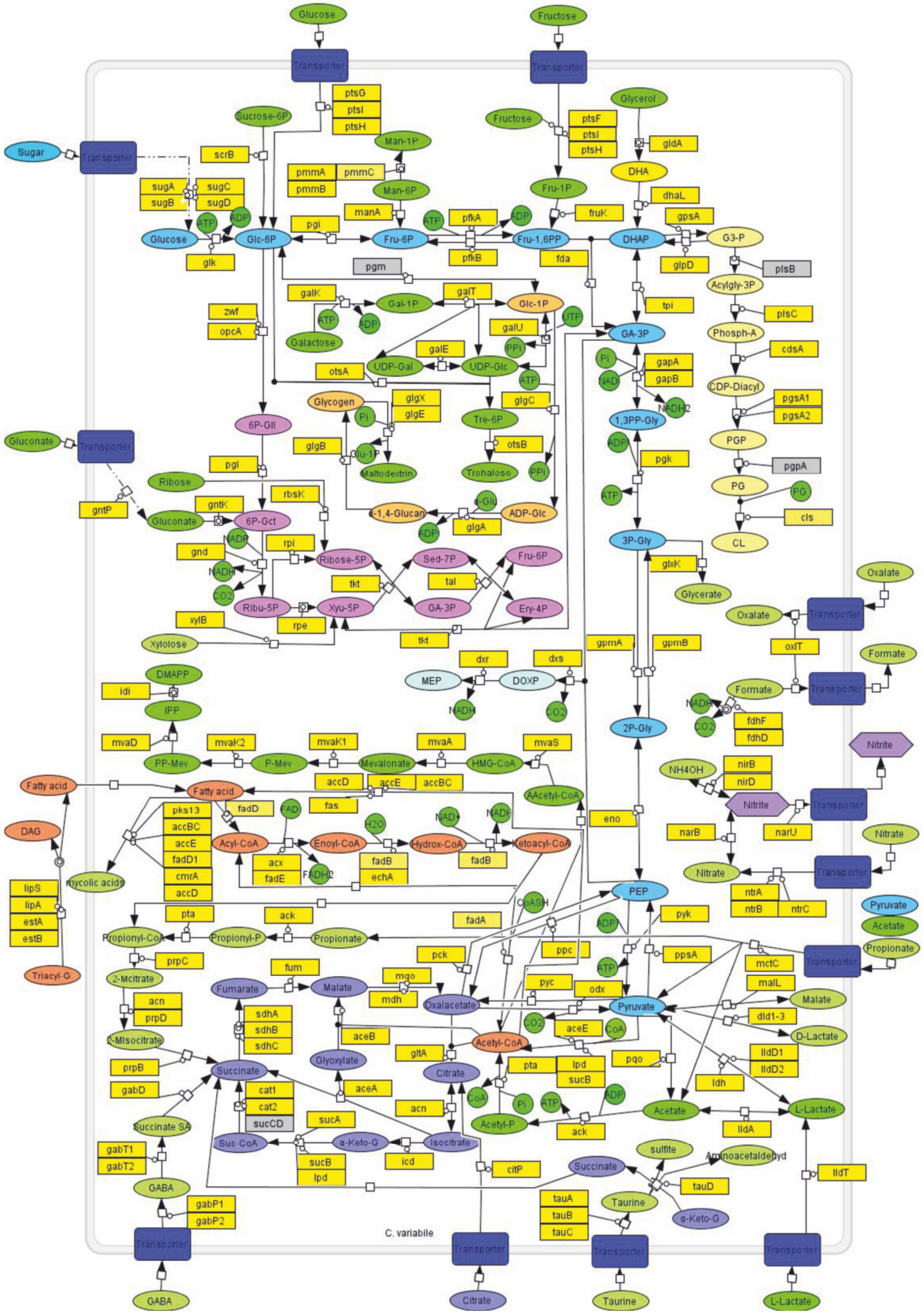

Glucose
Transporter
Fructose
Transporter
ptsG
ptsI
ptsH
Sucrose-6P
Glycerol
Fructose
ptsF
ptsI
ptsH
gldA
Sugar
Transporter
scrB
Man-1P
pmmA
pmmB
pmmC
Man-6P
DHA
sugA
sugB
sugC
sugD
Fru-1P
dhaL
fruK
ATP
ADP
manA
gpsA
Glucose
Glc-6P
pgi
Fru-6P
pfkA
pfkB
Fru-1,6PP
DHAP
G3-P
glk
pgm
fda
glpD
plsB
Acylgly-3P
galK
galT
Gal-1P
Glc-1P
tpi
plsC
zwf
opcA
Galactose
UTP
galU
GA-3P
Phosph-A
cdsA
galE
PPi
UDP-Gal
UDP-Glc
otsA
gapA
gapB
Pi
NAD
NADH2
CDP-Diacyl
pgsA1
pgsA2
Glycogen
glgX
glgE
glgC
Tre-6P
6P-Gll
1,3PP-Gly
PGP
glgB
Glu-1P
Maltodextrin
otsB
Trehalose
pgk
pgpA
Gluconate
Transporter
pgl
Ribose
PG
rbsK
cls
gntK
gntP
Gluconate
6P-Gct
α-1,4-Glucan
α-Glu
ADP-Glc
glgA
CL
NADP
rpi
gnd
Ribose-5P
Sed-7P
Fru-6P
3P-Gly
NADH
tkt
tal
glxK
CO2
Ribu-5P
rpe
Xyu-5P
GA-3P
Ery-4P
Glycerate
Oxalate
xylB
Oxalate
Transporter
Xylose
tkt
gpmA
gpmB
oxlT
Formate
DMAPP
dxr
dxs
MEP
DOXP
Formate
Transporter
idi
IPP
NADH
CO2
fdhF
fdhD
2P-Gly
mvaD
mvaK2
mvaK1
mvaA
mvaS
Nitrite
PP-Mev
P-Mev
Mevalonate
HMG-CoA
nirB
nirD
NH4OH
Fatty acid
Fatty acid
accD
accE
accBC
AAcetyl-CoA
Nitrite
Transporter
fas
fadD
pks13
FAD
eno
DAG
accBC
accE
fadD1
cmrA
accD
Acyl-CoA
Enoyl-CoA
H2O
NAD+
Hydrox-CoA
NADH
Ketoacyl-CoA
narB
narU
Nitrate
mycolic acids
acx
fadE
FADH2
fadB
echA
fadB
Nitrate
Transporter
lipS
lipA
estA
estB
ntrA
ntrB
ntrC
PEP
pta
ack
CoASH
fadA
Pyruvate
Acetate
Propionyl-CoA
Propionyl-P
Propionate
ADP
pyk
Propionate
Transporter
prpC
Triacyl-G
2-Mcitrate
pck
ppc
ppsA
mctC
fum
ATP
malL
mqo
pyc
Fumarate
Malate
mdh
odx
Malate
acn
prpD
Oxalacetate
Pyruvate
dld1-3
sdhA
sdhB
sdhC
CO2
aceE
CoA
D-Lactate
2-MIsocitrate
aceB
Acetyl-CoA
lldD1
lldD2
Glyoxylate
gltA
pqo
prpB
Succinate
Citrate
pta
lpd
sucB
ldh
gabD
cat1
cat2
sucCD
aceA
CoA
Pi
ATP
ADP
Acetate
L-Lactate
Succinate SA
sucA
acn
Acetyl-P
ack
lldA
Suc-CoA
α-Keto-G
Isocitrate
sulfite
Aminoacetaldehyd
gabT1
gabT2
sucB
lpd
icd
citP
Succinate
lldT
tauD
GABA
gabP1
gabP2
tauA
tauB
tauC
Taurine
α-Keto-G
C. variabile
Transporter
Transporter
Transporter
Transporter
GABA
Citrate
Taurine
L-Lactate

Additional file 1

Pathways involved in the central metabolism of *C. variabile* DSM 44702. The metabolic reconstruction was performed with manually curated pathway maps in conjunction with the bioinformatic tool CARMEN and the CellDesigner software. Abbreviations for metabolites are as follows: AAcetyl-CoA, Acetoacetyl-Coenzyme A; Acetyl-P, acetyl phosphate; Acyl-CoA, acyl-Coenzyme A; Acylgly-3P, 1-acyl-glycerol-3-phosphate; ADP, adenosine diphosphate; ATP, adenosine triphosphate; CDP-Diacyl, CDP-diacylglycerol; CL, cardiolipin; CoA/CoASH, Coenzyme A; DAG, diacylglycerol; DHAP, dihydroxyacetone phosphate; DMAPP, dimethylallyl diphosphate; DOXP, 1-deoxy-D-xylulose-5-phosphate; Enoyl-CoA, enoyl-Coenzyme A; Ery-4P, erythrose-4-phosphate; FAD, flavin-adenine-dinucleotide; Fru-1,6PP, fructose-1,6-bisphosphate; Fru-6P, fructose-6-phosphate; Gal-1P, galactose-1-phosphate; GA-3P, glyceraldehyde-3-phosphate; Glc-1P, glucose-1-phosphate; Glc-6P, glucose-6-phosphate; G3-P, glycerol-3-phosphate; HMG-CoA, β-hydroxy-β-methylglutaryl-Coenzyme A; Hydrox-CoA, hydroxyacyl-Coenzyme A; IPP, isopentenyl pyrophosphate; Ketoacyl-CoA, ketoacyl-Coenzyme A; Man-1P, mannose-1-phosphate; Man-6P, mannose-6-phosphate; MEP, 2-*C*-methyl-D-erythritol 4-phosphate; NAD/NADH2, nicotinamide adenine dinucleotide; NADP, nicotinamide adenine dinucleotide phosphate; PEP, phosphoenolpyruvate; PG, phosphoglycolate; PGP, 2-phosphoglycolate; Phosph-A, phosphatidate; Pi, phosphate; P-Mev, 5-phosphomevalonate; PPi, pyrophosphate; PP-Mev, 5-pyrophosphomevalonate; Propionyl-CoA, propionyl-Coenzyme A; Propionyl-P, propionyl phosphate; Ribose-5P, ribose-5-phosphate; Ribu-5P, ribulose-5-phosphate; Sed-7P, sedoheptulose-7-phosphate; Suc-CoA, succinyl-Coenzyme A; Tre-6-P, trehalose-6-phosphate; Triacyl-G, triacylglycerol; UDP-Gal, UDP-galactose; UDP-Glc, UDP-glucose; Xyu-5P, xylulose-5-phosphate; 1,3PP-Gly, 1,3-bisphosphoglycerate; 2-Mcitrate, 2-methylcitrate; 2-Misocitrate, 2-methylisocitrate; 2P-Gly, 2-phosphoglycerate; 3P-Gly, 3-phosphoglycerate; 6P-Gct, 6-phosphogluconate; 6P-Gll, 6-phosphogluconolactone; α-keto-G; α-ketoglutarate.
