# Supplementary material for: Complete genome sequence of Corynebacterium variabile DSM 44702 isolated from the surface of smear-ripened cheeses and insights into cheese ripening and flavor generation
Source: BMC Genomics. 2011 Nov 3;12:545. doi: 10.1186/1471-2164-12-545 (PMC3219685; doi:10.1186/1471-2164-12-545)
Supplement: Additional file 2 — Pathways involved in the biosynthesis of vitamins and cofactors by C. variabile DSM 44702. The PDF contains a reconstructed pathway map of vitamin and cofactor biosynthesis. [file 1471-2164-12-545-S2.PDF]

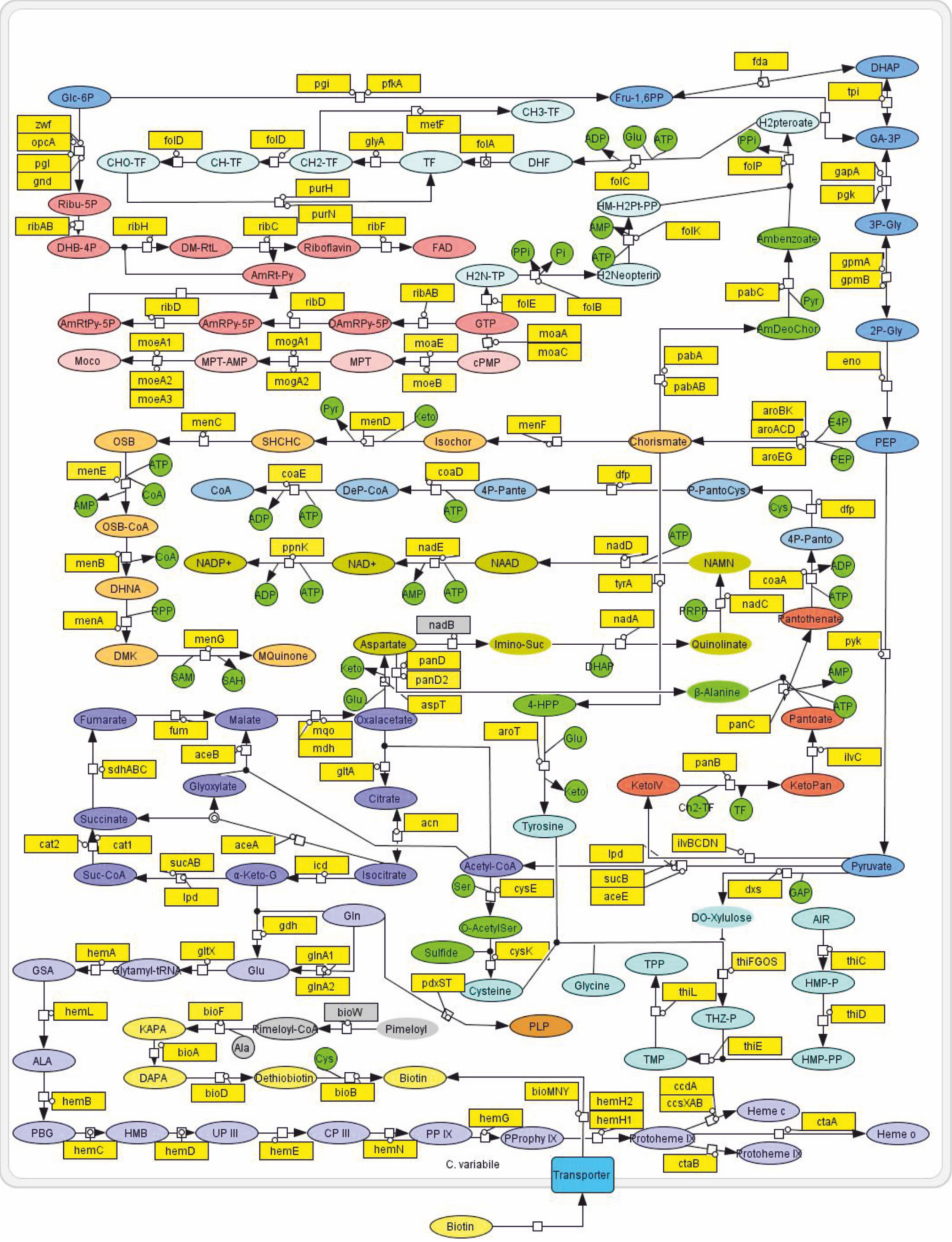
fda
DHAP
pgi
pfkA
Glc-6P
Fru-1,6PP
tpi
CH3-TF
metF
H2pteroate
GA-3P
zwf
opcA
pgl
gnd
folD
folD
glyA
folA
ADP
Glu
ATP
PPi
CHO-TF
CH-TF
CH2-TF
TF
DHF
folP
gapA
pgk
folC
purH
HM-H2Pt-PP
Ribu-5P
purN
ribAB
ribH
ribC
ribF
AMP
folK
Ambenzoate
3P-Gly
DHB-4P
DM-RtL
Riboflavin
FAD
PPi
Pi
ATP
gpmA
gpmB
AmRt-Py
H2N-TP
H2Neopterin
ribAB
folE
folB
pabC
Pyr
ribD
ribD
AmRtPy-5P
AmRPy-5P
OAmRPy-5P
GTP
AmDeoChor
2P-Gly
moaA
moaC
moeA1
mogA1
moaE
Moco
MPT-AMP
MPT
cPMP
pabA
eno
moeA2
moeA3
mogA2
moeB
pabAB
menC
menD
Pyr
Keto
menF
aroBK
aroACD
aroEG
E4P
OSB
SHCHC
Isochor
Chorismate
PEP
PEP
menE
ATP
CoA
AMP
coaE
coaD
dfp
CoA
DeP-CoA
4P-Pante
P-PantoCys
ADP
ATP
ATP
Cys
dfp
OSB-CoA
4P-Panto
ppnK
nadE
nadD
ATP
menB
CoA
NADP+
NAD+
NAAD
NAMN
ADP
ADP
ATP
AMP
ATP
tyrA
coaA
ATP
DHNA
nadC
PRPP
Pantothenate
menA
RPP
nadB
nadA
DMK
menG
MQuinone
Aspartate
Imino-Suc
Quinolinate
pyk
SAM
SAH
panD
panD2
DHAP
Keto
AMP
Glu
β-Alanine
aspT
4-HPP
ATP
Fumarate
Malate
Oxalacetate
fum
mqo
mdh
aroT
panC
Pantoate
Glu
aceB
ilvC
sdhABC
gltA
panB
Glyoxylate
Citrate
KetoIV
KetoPan
Keto
Ch2-TF
TF
Succinate
acn
Tyrosine
cat2
cat1
aceA
ilvBCDN
sucAB
icd
lpd
Acetyl-CoA
Pyruvate
Suc-CoA
α-Keto-G
Isocitrate
sucB
aceE
lpd
Ser
cysE
dxs
GAP
Gln
DO-Xylulose
AIR
gdh
O-AcetylSer
hemA
gltX
glnA1
Sulfide
cysK
thiFGOS
thiC
GSA
Glytamyl-tRNA
Glu
glnA2
TPP
Glycine
HMP-P
pdxST
Cysteine
thiL
hemL
bioF
bioW
PLP
THZ-P
thiD
KAPA
Pimeloyl-CoA
Pimeloyl
Ala
bioA
thiE
ALA
TMP
HMP-PP
Cys
DAPA
Dethiobiotin
Biotin
bioD
bioB
ccdA
ccsXAB
bioMNY
hemB
hemH2
hemH1
Heme c
hemG
ctaA
PBG
HMB
UP III
CP III
PP IX
PProphy IX
Protoheme IX
Heme o
hemC
hemD
hemE
hemN
Protoheme IX
ctaB
C. variabile
Transporter
Biotin

Additional file 2

Pathways involved in the biosynthesis of vitamins and cofactors by *C. variabile* DSM 44702. The metabolic reconstruction was performed with manually curated pathway maps in conjunction with the bioinformatic tool CARMEN and the CellDesigner software. Abbreviations for metabolites are as follows: ADP, adenosine diphosphate; AIR, 5-aminoimidazole ribonucleotide; ALA, δ-aminolevulinate; Ala, alanine; AmRPy-5P, 5-amino-6-ribosylamino-2,4-pyrimidinedione 5'-phosphate; AmRt-Py, 5-amino-6-ribitylamino-2,4-pyrimidinedione; AmRtPy-5P, 5-amino-6- ribitylamino-2,4-pyrimidinedione 5'-phosphate; ATP, adenosine triphosphate; CHO-TF, 10-formyl-tetrahydrofolate; CH-TF, 5-methyl-tetrahydrofolate; CH2-TF; 5,10-methylene-tetrahydrofolate; CH3-TF, methenyl-tetrahydrofolate; CoA, Coenzyme A; CP III, coproporphyrin III; cPMP, cyclic pyranopterin monophosphate; Cys, cysteine; DAmRPy-5P, 2,5-diamino-6-ribosylamino-4-pyrimidinone 5'-phosphate; DAPA, 7,8-diaminopelargonate; DeP-CoA, dephosphocoenzyme A; DHAP, dihydroxyacetone phosphate; DHB-4P, L-3,4-dihydroxy-2-butanone 4-phosphate; DHF, dihydrofolate; DHNA, 1,4-dixydroxy-2-naphthoic acid; DMK, demethylmenaquinone; DM-RtL, 6,7-dimethyl-8-ribityllumazine; FAD, flavin-adenine-dinucleotide; Fru-1,6PP, fructose-1,6-bisphosphate; GA-3P/GAP, glyceraldehyde-3-phosphate; Glc-6P, glucose-6-phosphate; Gln, glutamine; Glu, glutamic acid; GSA, glutamate-1-semialdehyde; HMB, hydroxymethylbilane; HM-H2Pt-PP, 6-hydroxymethyl-H_2pterin pyrophosphate; HMP-P, 4-amino-2-methyl-5-phosphomethylpyrimidine; HMP-PP, 4-amino-2-methyl-5-diphosphomethylpyrimidine; H2neopterin, dihydroneopterin; H2N-TP, H_2neopterin triphosphate; H2pteroate, dihydropteroate; Imino-Suc, α-iminosuccinate; Isochor, isochorismate; KAPA, 7-keto-8-aminopelargonate; KetoIV, α-ketoisovalerate; KetoPan, α-ketopantoate; Moco, molybdenum cofactor; MPT, molybdopterin; MPT-AMP, molybdopterin adenosine monophosphate; MQuinone, menaquinone; NAAD, Nicotinic acid adenine dinucleotide; NAD, nicotineamide adenine dinucleotide; NADP, nicotineamide adenine dinucleotide phosphate; NAMN, nicotinic acid mononucleotide; OSB, *o*-succinylbenzoic acid; OSB-CoA, *o*-succinylbenzoyl-Coenzyme A; PEP, phosphoenolpyruvate; PGB, probilinogen; Pi, phosphate; PLP, pyridoxal-5-phosphate; P-PantoCys, 4'-phosphopantothenoylcysteine; PPi, pyrophosphat; PP IX, protoporphyrinogen IX; Pprophy IX, protoporphyrin IX; PRPP, phosphoribosyl pyrophosphate; Ribu-5P, ribulose-5-phosphate; SAH, *S*-adenosylhomocysteine; SAM, *S*-adenosylmethionine; SHCHC, 2-syccinyl-6-hydroxy-2,4-cyclohexadiene-1-carboxylate; Suc-CoA, succinyl-Coenzyme A; TF, tetrahydrofolate; THZ-P, 5-(2-hydroxyethyl)-4-methylthiazole; TMP, thiamine phosphate; TPP, thiamine pyrophosphate; UP III, uroporphyrinogen III; 2P-Gly, 2-phosphoglycerate; 3P-Gly, 3-phosphoglycerate; 4P-Pante, 4'-phosphopantetheine; 4P-Panto, 4'-phosphopantothenate; α-keto-G; α-ketoglutarate.
