# Supplementary material for: Complete genome sequence of Corynebacterium variabile DSM 44702 isolated from the surface of smear-ripened cheeses and insights into cheese ripening and flavor generation
Source: BMC Genomics. 2011 Nov 3;12:545. doi: 10.1186/1471-2164-12-545 (PMC3219685; doi:10.1186/1471-2164-12-545)
Supplement: Additional file 3 — Pathways involved in amino acid metabolism of C. variabile DSM 44702. The PDF contains a reconstructed pathway map of amino acid biosynthesis and amino acid transport. [file 1471-2164-12-545-S3.PDF]

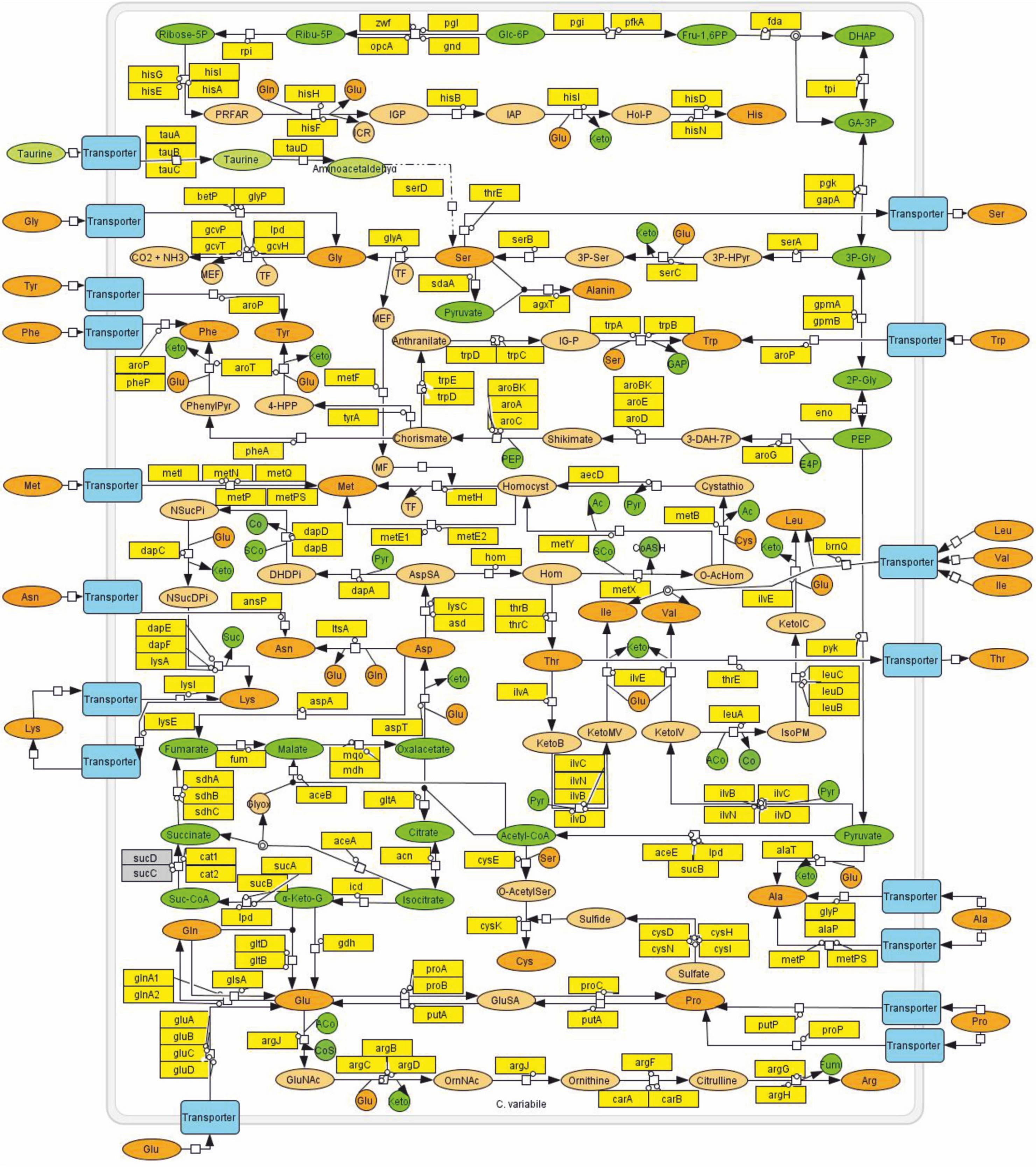

Ribose-5P
Ribu-5P
zwf
pgl
opcA
gnd
Glc-6P
pgi
pfkA
Fru-1,6PP
fda
DHAP
rpi
hisG
hisE
hisI
hisA
Gln
hisH
Glu
hisB
hisI
hisD
tpi
PRFAR
hisF
ICR
IGP
IAP
Hol-P
His
hisN
Glu
Keto
GA-3P
Taurine
Transporter
tauA
tauB
tauC
Taurine
tauD
Aminoacetaldehyd
serD
thrE
pgk
gapA
Transporter
Ser
Gly
Transporter
betP
glyP
gcvP
gcvT
lpd
gcvH
glyA
CO2 + NH3
Gly
Ser
serB
3P-Ser
Keto
Glu
serC
3P-HPyr
serA
3P-Gly
MEF
TF
TF
sdaA
agxT
Alanin
Tyr
Transporter
aroP
Pyruvate
gpmA
gpmB
Phe
Transporter
Phe
Tyr
MEF
Anthranilate
IG-P
trpA
trpB
Trp
Transporter
Trp
Keto
Keto
trpD
trpC
Ser
GAP
aroP
aroP
pheP
Glu
aroT
Glu
metF
trpE
trpD
aroBK
aroA
aroC
aroBK
aroE
aroD
2P-Gly
PhenylPyr
4-HPP
tyrA
eno
Chorismate
Shikimate
3-DAH-7P
PEP
pheA
PEP
aroG
E4P
MF
Met
Transporter
metI
metN
metQ
Met
metP
metPS
TF
metH
Homocyst
aecD
Cystathio
Ac
Pyr
NSucPi
Co
dapD
dapB
metE1
metE2
metB
Ac
Leu
Glu
SCo
metY
CoASH
Cys
dapC
Keto
Pyr
hom
SCo
Keto
brnQ
Leu
Transporter
Val
DHDPi
AspSA
Hom
O-AcHom
Ile
dapA
metX
Asn
Transporter
NSucDPi
ansP
ilvE
Glu
Ile
Val
lysC
asd
thrB
thrC
KetoIC
dapE
dapF
lysA
Suc
ltsA
Asn
Asp
Thr
Keto
pyk
Transporter
Thr
Glu
Gln
ilvE
thrE
leuC
leuD
leuB
lysI
Keto
Transporter
Lys
Lys
aspA
ilvA
Glu
lysE
Glu
aspT
KetoMV
KetoIV
leuA
IsoPM
KetoB
Transporter
Fumarate
Malate
mqo
mdh
Oxalacetate
fum
ACo
Co
sdhA
sdhB
sdhC
ilvC
ilvN
ilvB
ilvD
aceB
Glyox
gltA
ilvB
ilvC
Pyr
Pyr
ilvN
ilvD
Succinate
Citrate
Acetyl-CoA
Pyruvate
aceA
sucD
sucC
cat1
cat2
acn
cysE
Ser
aceE
lpd
sucB
alaT
sucA
Keto
Glu
sucB
icd
O-AcetylSer
Ala
Transporter
Suc-CoA
α-Keto-G
Isocitrate
lpd
cysK
Sulfide
glyP
alaP
Ala
Gln
gltD
gltB
gdh
cysD
cysN
cysH
cysI
Cys
metP
metPS
Transporter
glnA1
glnA2
glsA
proA
proB
proC
Sulfate
Glu
GluSA
Pro
putA
putA
Transporter
gluA
gluB
gluC
gluD
ACo
Pro
putP
proP
argJ
CoS
Transporter
argB
argC
argD
argJ
argF
argG
Fum
GluNAc
OrnNAc
Ornithine
Citrulline
Arg
argH
Glu
Keto
carA
carB
C. variabile
Transporter
Glu

Additional file 3

Pathways involved in amino acid metabolism of *C. variabile* DSM 44702. The metabolic reconstruction was performed with manually curated pathway maps in conjunction with the bioinformatic tool CARMEN and the CellDesigner software. Abbreviations for metabolites are as follows: Ac, acetate; ACo, acetyl-Coenzyme A; Ala, alanine; Arg, arginine; Asn, asparagine; Asp, aspartic acid; AspSA, aspartate semialdehyde; CoA/CoASH, Coenzyme A; Cys, cysteine; Cystathio, L,L-cystathionine; DHAP, dihydroxyacetone phosphate; DHDPi, dihydrodipicolinate; E4P, erythrose-4-phosphate; Fru-1,6PP, fructose-1,6-bisphosphate; Fum, fumarate; GA-3P/GAP, glyceraldehyde-3-phosphate; Glc-6P, glucose-6-phosphate; Gln, glutamine; Glu, glutamic acid; GluNAc, *N*-acetylglutamate; GluSA, γ-glutamic semialdehyde; Gly, glycin; Glyox, glyoxalate; His, histidine; Hol-P, L-histidinol phosphate; Hom, homoserine; Homocyst, homocysteine; IAP, imidazole acetol phosphate; IGP, imidazole glycerol phosphate; IG-P, indole glycerol phosphate; Ile, isoleucine; IsoPM, α-isopropylmalate; KetoB, α-ketobutyrate; KetoIC, α-ketoisocaproate; KetoIV, α-ketoisovalerate; KetoMV, α-keto-β-methylvalerate; Leu, leucine; Lys, lysine; MEF, methylenetetrahydrofolate; Met, methionine; MF, methylene-tetrahydropteroyl; NSucDPi, *N*-succinyldiaminopimelate; NSucPi, *N*-succinyl-α-amino-ε-keto-pimelate; O-AcetylSer, *O*-acetylserine; O-AcHom, *O*-succinylhomoserine; OrnNAc, *N*-acetylornithine; PEP, phosphoenolpyruvate; Phe, phenylalanine; Phenylpyr, phenylpyruvate; PRFAR, phosphoribosyl formimino-5-aminoimidazole-4-carboxamide ribonucleotide; Pro, proline; Pyr, pyruvate; Ribose-5P, ribose-5-phosphate; Ribu-5P, ribulose-5-phosphate; Ser, serine; Suc-CoA/SCo, succinyl-Coenzyme A; TF, tetrahydrofolate; Thr, threonine; Trp, tryptophan; Tyr, tyrosine; Val, valine; 2P-Gly, 2-phosphoglycerate; 3-DAH-7P, 3-deoxy-D-arabino-heptulosonate-7-phosphate; 3P-HPyr, 3-phosphohydroxypyruvate; 3P-Gly, 3-phosphoglycerate; 3P-Ser, 3-phosphoserine; 4-HPP, 4-hydroxyphenylpyruvate;α-keto-G/Keto; α-ketoglutarate.
